# Impact of APOE genotype on prion-type propagation of tauopathy

**DOI:** 10.1186/s40478-022-01359-y

**Published:** 2022-04-19

**Authors:** Tristan Williams, Alejandra Jolie Ruiz, Angelica Maria Ruiz, Quan Vo, Wangchen Tsering, Guilian Xu, Karen McFarland, Benoit I. Giasson, Patrick Sullivan, David R. Borchelt, Paramita Chakrabarty

**Affiliations:** 1 Center for Translational Research in Neurodegenerative Disease, University of Florida, 1275 Center Drive, Gainesville, FL 32610 USA; 2 Department of Neuroscience, University of Florida, 1149 Newell Dr, Gainesville, FL 32610 USA; 3 Department of Neurology, University of Florida, Gainesville, FL 32610 USA; 4 McKnight Brain Institute, University of Florida, 1275 Center Drive, BMS J484, Gainesville, FL 32610 USA; 5 Department of Medicine, Duke University, Durham, NC 27710 USA; 6 McKnight Brain Institute, University of Florida, 1275 Center Drive, BMS J499, Gainesville, FL 32610 USA

**Keywords:** APOE, Prion, Phosphorylated tau, Tangle

## Abstract

Apolipoprotein (APOE) is a major risk factor of Alzheimer’s disease (AD), with the E2, E3 and E4 isoforms differentially regulating the burden of AD-associated neuropathologies, such as amyloid β and tau. In AD, pathological tau is thought to spread along neuroanatomic connections following a prion-like mechanism. To provide insights into whether APOE isoforms differentially regulate the prion properties of tau and determine trans-synaptic transmission of tauopathy, we have generated human P301S mutant tau transgenic mice (PS19) that carry human APOE (APOE2, APOE3 or APOE4) or mouse Apoe allele. Mice received intrahippocamal injections of preformed aggregates of K18-tau at young ages, which were analyzed 5 months post-inoculation. Compared to the parental PS19 mice with mouse Apoe alleles, PS19 mice expressing human APOE alleles generally responded to K18-tau seeding with more intense AT8 immunoreactive phosphorylated tau athology. APOE3 homozygous mice accumulated higher levels of AT8-reactive ptau and microgliosis relative to APOE2 or APOE4 homozygotes (E3 > E4~2). PS19 mice that were heterozygous for APOE3 showed similar results, albeit to a lesser degree. In the timeframe of our investigation, we did not observe significant induction of argentophilic or MC1-reactive neurofibrillary tau tangle in PS19 mice homozygous for human APOE. To our knowledge, this is the first comprehensive study in rodent models that provides neuropathological insights into the dose-dependent effect of APOE isoforms on phosphorylated tau pathology induced by recombinant tau prions.

**Supplementary Information:**

The online version contains supplementary material available at 10.1186/s40478-022-01359-y.

## Introduction

Alzheimer’s disease (AD) and related dementias are characterized by stereotypic progression of phosphorylated tau (ptau) along neuropathologically connected pathways [1, 2]. Whether the induction and transmission of tau pathology across different brain regions is regulated by genetic risk factors is debated.

Apolipoprotein E (APOE), a lipoprotein that functions as a cholesterol carrier, is a major genetic risk factor for AD [3, 4]. It exists in three major isoforms, APOE2, APOE3 and APOE4, that differ in sequence at two positions. While APOE3 is the predominant form and considered the neutral allele, APOE4 increases the risk of AD and APOE2 is protective. Much of APOE4’s function, as related to AD risk, has been experimentally related to cardiovascular homeostasis, blood brain barrier integrity and regulation of amyloid β (Aβ) deposition [5], while its role in regulating tauopathy is less studied. Human studies indicate that APOE4 is related to severe tauopathy in AD only in the presence of high levels of Aβ [6, 7]. Moreover, APOE4 patients are less vulnerable to primary age-related tauopathy (PART), a form of Aβ-independent tauopathy with limited cognitive impairment [8, 9]. The relationship between APOE isoforms and Fronto-temporal dementias (FTD), another tauopathy characterized by tau in the absence of Aβ but showing high degree of cognitive impairment, is more obscure [10–12]. While there is no clear association between FTD risk and APOE genotype, certain subsets in the FTD spectrum are associated with APOE2 [13] whereas other studies have found that APOE4 correlates with disease risk [14]. Together, these studies indicate that the role of APOE isoforms on tau pathogenesis is unresolved.

Recent studies in transgenic models of tauopathy have also not produced clear-cut associations (reviewed in [5]). In a transgenic rodent model of tauopathy, APOE4 was shown to trigger tau-associated neurodegeneration [15–17], while another report found that APOE2 exacerbates pathologic tau inclusions in a hyper-expression model of tau [10]. Thus, which specific APOE allele directly underlies the induction and subsequent transmission of tauopathy is uncertain. Given that tauopathy is broadly believed to be spread in a prionoid manner [18], our primary goal here was to characterize the induction and early stage progression of tau pathology in APOE mice seeded with recombinant tau prions.

Prionoid transmission of tauopathy can be initiated in rodent brains by the delivery of seeds formed of aggregated recombinant tau protein or homogenates from patient brain [19–21]. We and others have shown that pre-formed aggregated forms of K18 tau, containing the four microtubule binding domains, can induce AD-typical tau pathology when injected in the hippocampus of the PS19 model of transgenic mice expressing human P301S mutant tau [22]. Here, we created bigenic mouse models of Line PS19 mice bearing one or two alleles of human APOE genes replacing the mouse Apoe gene. We injected recombinant K18-tau aggregates into the hippocampus and assessed the ipsilateral and contralateral transmission of tauopathy in these mice after 5 months. In the K18-tau aggregate seeded cohort, we found that PS19 mice bearing APOE3 allele showed robust forebrain induction of ptau and microgliosis, compared to mice bearing APOE2 or APOE4 alleles (E3 > E4~E2). Generally, the human APOE bearing mice had higher ptau burden compared to PS19 mice with murine Apoe. Together, our study indicates that the APOE3 isoform increases the severity of tau hyperphosphorylation in response to tau prions.

## Methods

### Mice and study design

Mouse husbandry and experimental procedures were performed in accordance with the protocols and policies approved by the Institutional Animal Care and Use Committee at the University of Florida. All mice were maintained under a 12-h light/dark cycle and had access to water and food ad libitum. The mice were maintained under specific pathogen-free conditions and all surgeries were done under anesthesia using sterile conditions by a trained surgeon. No adverse events like sudden death or inability to recover from anesthesia were observed following surgery. PS19 mice were obtained from Jackson Labs and maintained on a B6/C3H background as heterozygotes for P301S tau transgene, developing age-progressive tauopathy and hindlimb paralysis at 9–12 months of age [23]. APOE targeted replacement (TR) mice were obtained from Duke University and were maintained as homozygotes on C57BL6 background [24–26]. PS19 mice were mated with APOE mice to produce N1 cohorts that were heterozygous for APOE. N2 cohorts of PS19 mice that are heterozygous or homozygous for APOE were generated by back-crossing the N1 generation mice with corresponding APOE homozygous mice. N1 and N2 cohorts from each APOE genotype (E2, E3, E4) were set up to be injected with K18-tau fibrils or phosphate buffered saline (PBS) into the left hippocampus (Additional file 12: Table S1). At endpoint, all euthanasia was performed with intracardiac perfusion of cold PBS containing heparin and the brains were fixed in 10% normal buffered formalin (Fisher Scientific). The fixed brains were then sliced coronally at the indicated bregma locations and processed for paraffin embedding.

### Hippocampal stereotactic injections

Mice were aged to 2.5 months and unilaterally injected into the left hippocampus (coordinates from Bregma: A/P − 2.2, L − 1.6, D/V − 1.2) with K18-tau aggregates. K18-tau aggregates were generated and sonicated as described earlier [22]. Aggregates (3 µl of 1 mg/ml) were injected into the brain at 0.3 µl per minute. Control mice were injected with sterile PBS in the hippocampus. Mice were allocated randomly to each experimental cohort. Injected mice were aged for 5 months and analyzed at 7.5 months of age. The mouse numbers for the different injection groups are shown in Additional file 12: Table S1.

### Immunohistochemical analysis of brain sections

Paraffin embedded slides with coronal brain sections were deparaffinized and probed with primary antibodies (Additional file 12: Table S2) as described before [22]. For antigen retrieval, slides receiving AT8, PHF1, MC1 and GFAP antibodies were steamed in water at high pressure for 15 min, while Iba-1, Tmem119, and CD68 antibody treated slides were steamed in citrate buffer pH 6.0 (Target Retrieval Solution, Dako). Slides were incubated in 3% hydrogen peroxide (Fisher Scientific) for 20 min to block endogenous peroxidase activity and then washed three times in PBS for 5 min each. Slides were then blocked in 2% FBS (Hyclone, GE) for 45 min before incubating in primary antibody diluted in block solution overnight at 4 °C (Additional file 12: Table S2). The following day, slides were washed and appropriate secondary antibody (ImmPRESS Polymer Reagent, Vector Labs) was applied for 30 min at room temperature. Following PBS washes, color was developed using 3,3′-diaminobenzidine (Vector DAB, Vector Labs) and slides were counterstained with haematoxylin (Vector Labs). Next, brain sections were dehydrated in a series of ethanol, cleared in xylene, mounted in Cytoseal-60 media (Fisher Scientific) and coverslipped.

### Gallyas silver impregnation protocol

Gallyas silver impregnation protocol was done as described earlier [27]. Briefly, sections were rehydrated and incubated for 5 min in 5% periodic acid. Following two 5 min washes in water, sections were incubated in alkaline silver iodide solution for 1 min and then washed in 0.5% acetic acid for 10 min. Next, slides were placed in developer solution for ~ 5 min. Following development, slides were washed in 0.5% acetic acid for 3 min and then water for 5 min. The next steps were as follows: 5 min incubation in 0.1% gold chloride, rinsing in dH20, 5 min incubation in 1% sodium thiosulphate solution, and rinsing in tap water. Counterstaining was done with hematoxylin, and sections were dehydrated, cleared, and mounted. Analysis of Gallyas staining was done by manual counting of silver positive neurons.

### Analysis of histochemical images

Initial slides were stained with haematoxylin and eosin, their location identified using Paxinos and Franklin’s mouse brain atlas and further slides were prepared according to desired bregma locations. Immunostained images were captured using a Scanscope XT image scanner (Aperio, Vista, CA, USA). Percent immunoreactivity was computed using the Aperio Positive Pixel Count program which quantifies the area and intensities of staining based on user‐defined values for color and intensity thresholds (Aperio, Vista, CA, USA). Regions of interest stained with DAB were selected and then quantified by using custom-generated algorithms after defining the hue, saturation and intensity values corresponding to brown color. Slides were stained on the same day using identical batches of buffers and reagents to minimize variability. Areas with artefactual staining (folded or torn areas) were excluded using negative pen tool annotation. For pixels corresponding to the specified color, the algorithm counts the number and intensity sum in each intensity range. Pixels which are stained, but do not correspond to the specified color, are considered negative stained pixels. These pixels are also counted to determine the fraction of positive to total stained pixels. The output results, expressed as percent immunoreactivity, is equal to total positive pixel counts normalized to total area. Ipsilateral and contralateral hemispheres (based on injection site) were identified and % immunoreactivity from cortex and hippocampus were quantified. The data is shown as the average % immunoreactivity ± S.E.M. per group. A summary of antibody staining data is shown in Additional file 12: Table S3. Statistical comparisons were conducted using 1-way ANOVA (GraphPad Prism 7).

### Brain propagation analysis and heatmap creation

We generated semi-quantitative heat maps to assess spatial distribution of immunoreactivity using 3–4 individual samples corresponding to the indicated area of interest. Semi-quantitative analysis was performed on antibody-stained sections that was scored by two blinded individuals on a scale from 0 to 3 (0: no pathology; 1: low pathology; 2: medium pathology; 3: high pathology) at the assigned coronal bregma level (− 2.03 was designated as injection level) (Additional file 1: Fig. S1). After the separate brain regions were scored for each individual mouse, the average values were imported into Microsoft Excel following a protocol described earlier [22]. The coronal mouse brain illustrations were adapted from the Allen Mouse Brain Atlas. The schematic for the connectivity map was derived from the Allen Brain Atlas: Mouse Brain Connectivity Atlas [28].

### RNA isolation from formalin fixed paraffin-embedded brain sections

Nine 20 µm thick sections per brain were placed in tissue cassettes and deparaffinized through a cycle of incubation in xylene for two times 10 min each, 100% EtOH for two times 10 min each, 90% EtOH for 10 min, and 70% EtOH for 10 min followed by a rinse in water. Brain sections that contained hippocampus and cortex proximal to injection site was retrieved for RNA extraction. RNA was extracted using the High Pure FFPET RNA Isolation Kit (Roche) following the manufacturer’s protocols. RNA concentration was calculated with the Nanodrop system and RNA quality was evaluated with the Bioanalyzer system using RNA nano chips and the DV200 analysis module according to manufacturer’s instructions.

### Nanostring nCounter analysis

RNA samples were diluted to 20 ng/µl before undergoing hybridization with the capture and reporter probe from the nCounter Neuropathology CodeSet for 16–24 h at 65 °C. Then, the samples were transferred to the Nanostring Prep station where excess probes were removed. The purified samples were then bound, immobilized, and aligned on the imaging surface of the nCounter Cartridge followed by automated analysis by the Digital Analyzer. Data was normalized and analysed using the nSolver software. Graphs were drawn with ggraph v2.0.4 in R. p-values were adjusted for multiple comparisons.

### Statistics

Immunohistochemical data was analyzed using 1-way ANOVA unless otherwise indicated in figure legend. Outliers were removed using Rout’s test with Q = 1%. A summary of APOE effects is summarized in Supplementary Table S3. All p values in the study are presented in Additional file 12: Tables S4-S7. The neuropathological scoring was assessed by two blinded observers individually and then collated for data analysis. All data was assembled using Adobe Photoshop Elements.

## Results

### Generation of PS19 mice carrying human APOE alleles

Mice carrying humanized APOE alleles [24–26] were bred with P301S tau transgenic Line PS19 mice [23] to generate tau transgenic mice that were homozygous (PS/E2H, PS/E3H and PS/E4H) or heterozygous (PS/E2h, PS/E3h and PS/E4h) for human APOE. The PS19 mice used in this study were maintained as C3H/B6 hybrids whereas the APOE mice were congenic on the C57BL/6 J strain. Thus, the first generation of progeny resulting from this cross, consisting of mice heterozygous for tau and APOE were B6N1. Subsequently these B6N1 mice were back-crossed to parental APOE strains resulting in tau mice heterozygous or homozygous for the APOE on a B6N2 background. We confirmed that levels of tau transgene expression were similar in PS19 mice homozygous or heterozygous for each APOE (B6N2) relative to parental PS19 mice bearing two alleles of mouse Apoe (Additional file 2: Fig. S2a-b). We also confirmed the presence of specific human alleles by sequencing of tail DNA (data not shown) or using an antibody that specifically recognizes human APOE4 (Additional file 2: Fig. S2c-d). We next analyzed the presence of APOE levels in the PS19 mice homozygous for each APOE (B6N2) (Additional file 3: Fig. S3). Using RIPA-soluble forebrain lysates, we observed that the level of APOE2 was higher than APOE3 and APOE4, both in the parental APOE mice as well as in the corresponding PS19xAPOE bigenic mice. This finding is consistent with previous observations that APOE2 levels are higher relative to APOE3 and APOE4 in human postmortem brain [29] and in plasma [30]. Within each APOE genotype, there was no difference in APOE levels between the tau transgenic and tau non-transgenic mice (Additional file 3: Fig. S3a).

We performed immunohistochemistry (IHC) in the spinal cord (Additional file 3: Fig. S3b) and brain (Additional file 3: Fig. S3c) to determine the distribution of APOE immunoreactivity. In the spinal cord, we observed diffuse parenchymal staining in the grey matter and white matter areas, but no staining in the spinal motoneurons was noted (Additional file 3: Fig. S3b). Representative immunohistochemistry data from different areas of the brains of the in PS19 mice homozygous for APOE reveals diffuse staining in the forebrain, with cellular staining resembling non-neuronal cells (Additional file 3: Fig. S3c). To confirm the cell-type specific expression patterns of APOE in PS19 mice homozygous for APOE, we conducted co-immunofluorescence using anti GFAP (astrocyte-specific) and anti Iba-1 (microglia-specific) antibodies. We identified APOE within astrocytes of all the three APOE genotypes while APOE within microglial cells was detected at a lower frequency in all three genotypes (Additional file 3: Fig. S3d, arrows in ‘Merge’ panels).

### Induction of hippocampal tauopathy in K18-tau aggregate seeded PS19 mice homozygous for APOE

**Fig. 1:**
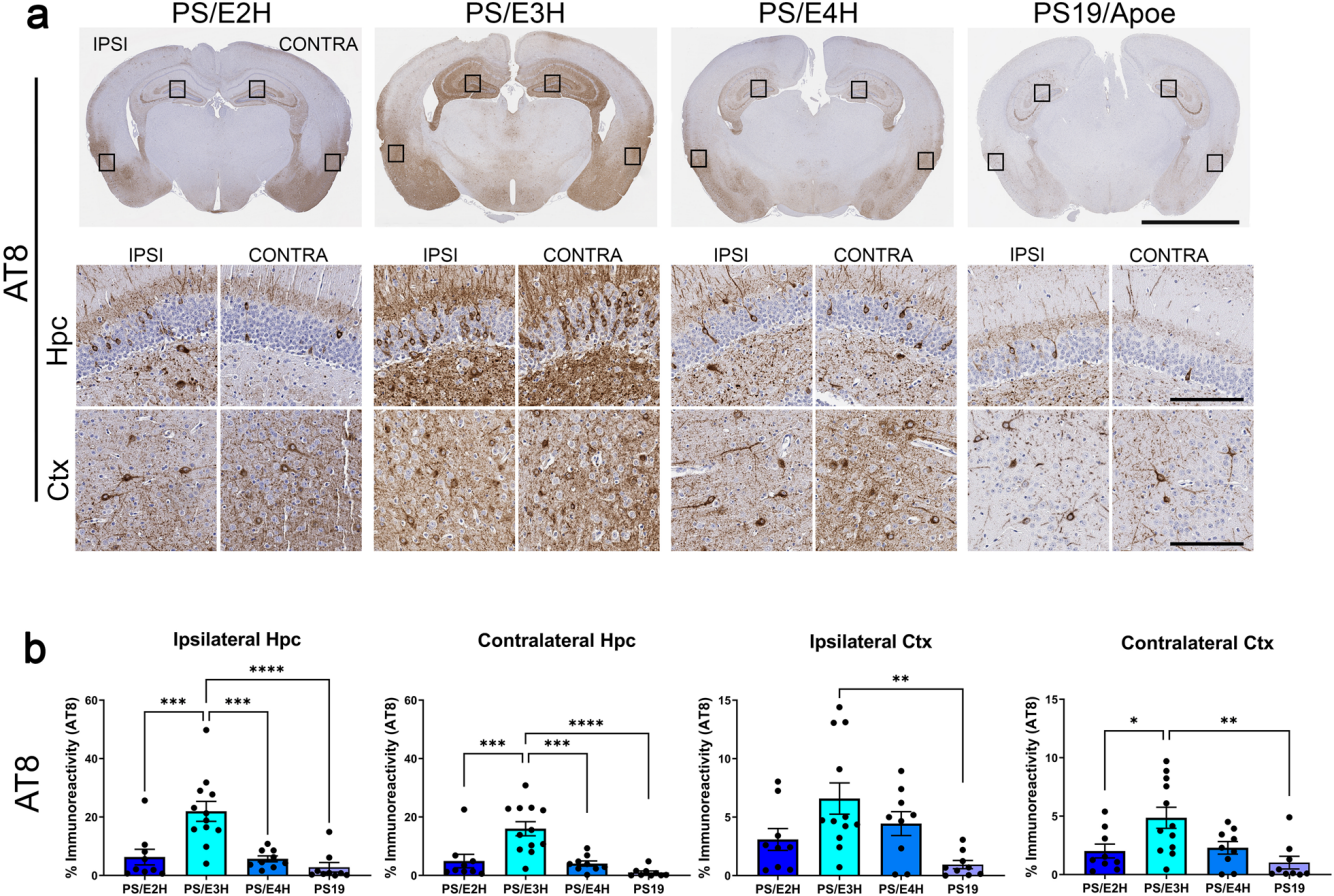
Accelerated induction of ptau pathology in PS/E3H mice seeded with K18-tau aggregates. K18-tau fibrils were injected into the left hippocampus of 2.5-month-old mice and aged for 5 months. 7.5-month-old mice were then analyzed using AT8 antibody. Representative images from the hippocampus and cortex of K18-tau aggregate injected hemisphere (ipsilateral, ‘IPSI’) and uninjected hemisphere (contralateral, ‘CONTRA’) showing ptau pathology in PS19 mice homozygous for APOE alleles (B6N2 generation) or PS19 mice carrying murine Apoe (**a**). Quantification of the antibody immunostaining is presented as % immunoreactivity in the cortex (Ctx) or hippocampus (Hpc) from ipsilateral and contralateral hemispheres around the injection site (**b**). Boxes in whole brain panel indicate selected areas used for high power zoomed panels. n = 9–12 mice/group. 1-way ANOVA *p < 0.05, **p < 0.01, ***p < 0.001, ****p < 0.0001. Scale bar: 3 mm (whole brain); 100 µm (hippocampus and cortex)

To investigate whether APOE alleles influence the prionoid induction and spread of tauopathy, we injected human K18-tau aggregates unilaterally into the left hippocampus of 2.5 month old PS/E2H, PS/E3H and PS/E4H mice and analyzed ptau burden after 5 months (Fig. 1). Parental PS19 mice (bearing murine Apoe) were also injected to assess the relative effect of mouse Apoe and human APOE on induction of tauopathy. Independently, PS/E2H, PS/E3H, PS/E4H and PS19 mice injected unilaterally with PBS in the hippocampus were treated as vehicle control group (Additional file 4: Fig. S4). These PBS injected mice showed equivalent levels of AT8 and PHF1 immunoreactivity, albeit with high inter-sample variability (Additional file 4: Fig. S4a-d). In the K18-tau injected cohorts, looking first at local ptau induction in hippocampus ipsilateral to the K18-tau injection site, we observed the highest levels of AT8-reactive ptau in PS/E3H mice relative to all other genotypes (*p* < 0.001 to *p* < 0.0001) (Fig. 1a, b). The induction of ptau in the contralateral hippocampus in PS/E3H mice was also more pronounced relative to all other genotypes (*p* < 0.001–0.0001) (Fig. 1b). In the ipsilateral cortex, PS/3EH mice showed statistically higher levels of ptau relative to parental PS19 mice (*p* < 0.01). Relative to PS/E2H and parental PS19 mice, the level of ptau reactivity in the contralateral cortex of PS/E3H mice was significantly higher (*p* < 0.05–0.01). In the spinal cords of these K18-tau seeded mice, we did not observe significantly accelerated induction of ptau pathology across the three APOE genotypes relative to PBS injected mice (Additional file 5: Fig. S5). This finding implies that within the timeframe examined, the induction of seeded tau pathology was predominantly confined in the brain. We also examined the level of PHF1 immunoreactive ptau in these cohorts (Additional file 6: Fig. S6a). Quantification of these data was more difficult due to high reactivity of the PHF1 antibody to endogenous murine tau, but notably within the ipsilateral hippocampus we observed that PS/E3H mice showed higher PHF1-reactive ptau burden compared to all other genotypes (*p* < 0.05–0.001) (Additional file 6: Fig. S6a-b). There was a suggestive trend observed in other brain regions of PS/E3H mice relative to the other PS19xAPOE cohorts. Collectively, these findings suggest that the local induction of ptau pathology by K18-tau seeding was more efficient in PS/E3H mice, with this ptau pathology then appearing to spread to the contralateral side.

We next examined the effect of hippocampal K18-tau seeding and PBS injection on induction of neurofibrillary tangle (NFT) pathology using Gallyas histological stain in different brain regions (Additional file 6: Fig. S6c-d). Compared to the PBS injected genotype-matched mice, the APOExPS19 mice showed higher levels of NFT (Additional file 6: Fig. S6c-e). However, total NFT levels in the K18-tau injected mice were not statistically different when compared to PBS injected genotyped-matched mice across the brain regions examined because of high inter-animal variability. Interestingly, in the parental PS19 mice, though the levels of NFT tau was overall lower, we observed that the K18-tau induced NFT count was significantly higher in the contralateral hippocampus and ipsilateral cortex compared to PBS-injected mice (Additional file 6: Fig. S6e). We further used antibody-based methods to confirm the burden of pre-tangle tau in K18-tau injected mice. Using immunostaining with MC1 (misfolded tau) or TauC3 (indicative of caspase-cleaved tau) antibodies, we found no differential induction across the three APOE genotypes (Additional file 7: Fig. S7). Thus, in spite of increased ptau burden relative to PS/E2H and PS/E4H mice, K18-tau aggregate seeded PS/E3H did not show robust acceleration of pre-tangle tau or NFT.

We then tested whether the outcome of tau seeding is dependent on APOE3 gene dosage (Additional file 8: Fig. S8). In the APOE heterozygous siblings of cohort described above (B6N2 generation), the PS/E3h mice showed higher AT8 burden in the ipsilateral and contralateral cortex compared to PS/E2h mice (*p* < 0.05), with a trend towards higher burden in the ipsilateral and contralateral hippocampus compared to PS/E2h and PS/E4h mice (Additional file 8: Fig. S8a-b). The PS/E3h mice also showed higher PHF1-tau immunoreactivity relative to PS/E2h mice (*p* < 0.05 in ipsilateral hippocampus) and relative to PS/E4h mice (*p* < 0.01 in ipsilateral cortex) (Additional file 8: Fig. S8c-d). These mice did not show any differential induction of MC1-positive pretangle tau based on their APOE status (Additional file 8: Fig. S8e-f), similar to their homozygous siblings.

In the B6N1 generation of heterozygous APOE mice, we found few differences in tau burden stratified by genotype (Additional file 9: Fig. S9). As before, we injected K18-tau aggregates or PBS in the left hippocampus of these mice at 2.5 months of age and analyzed these at 7.5 months. The PBS-injected unseeded controls did not show significant APOE-dependent effects on AT8 and PHF1 tau levels at this age (Additional file 9: Fig. S9a, b, e, f). AT8 immunostaining showed higher tau burden in the K18-tau seeded PS/E2h mice in the contralateral hippocampus (*p* < 0.05) relative to PS/E4h mice, while only a trend was noted in the ipsilateral hippocampus (Additional file 9: Fig. S9c, d). Similarly, PHF1 antibody also showed a similar outcome with PS/E2h mice showing higher tauopathy in the contralateral hippocampus (*p* < 0.01) and only a trend in ipsilateral hippocampus (*p* = 0.077) relative to PS/E4h mice (Additional file 9: Fig. S9 g, h). There was no change in tau staining in the ipsilateral cortex of these K18-tau seeded mice (Additional file 9: Fig. S9 d, h). We also did not observe any preferential induction of MC1 immunoreactive pre-tangle tau pathology in these mice (data not shown). Collectively, these findings from B6N2 (Fig. 1; Additional file 8: Fig. S8) and B6N1 (Fig. S9) mice suggest that there may be interactions between APOE gene dosage and background strain that modulate the influence of APOE on prion behavior of tau.

### Patterns of tauopathy spread in K18-tau aggregate seeded PS19 mice homozygous for human APOE

**Fig. 2:**
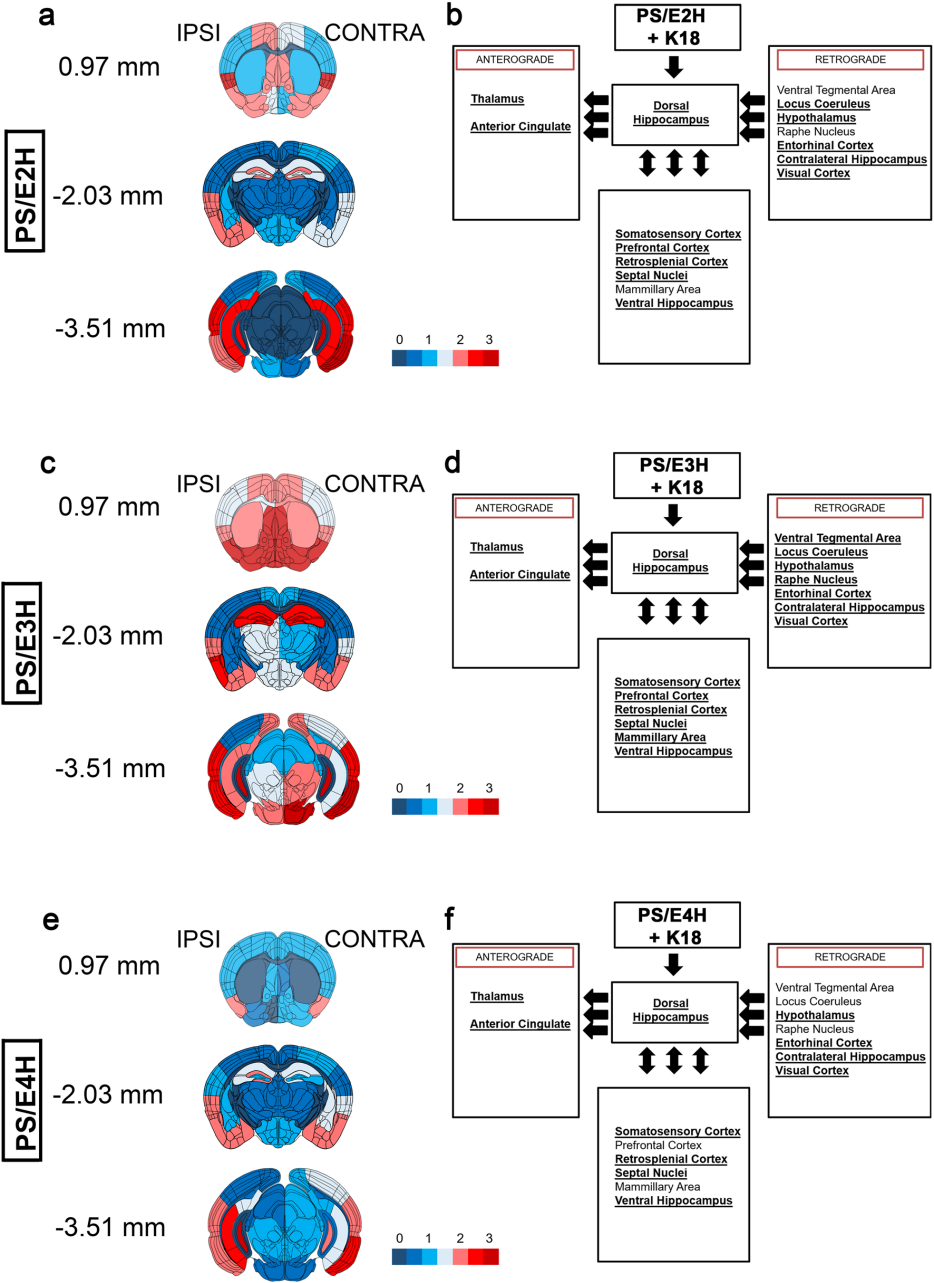
Relative abundance of ptau in hippocampus and neuroanatomically connected brain regions in K18-tau aggregate seeded PS19 mice carrying human APOE alleles. Semi-quantitative analyses of ptau transmission patterns along neuroanatomic pathways represented by AT8 immunostaining is shown from K18-tau aggregate seeded PS19 mice homozygous for human APOE alleles (B6N2 generation). Pathology severity was assigned scores on a scale of 0 (no pathology) to 3 (high pathology) and color-coded onto heat maps (a, c, e). The injected hemisphere (ipsilateral, ‘IPSI’) is shown on the left and non-injected (contralateral, ‘CONTRA’) hemisphere is depicted on the right for each heat map. Three coronal planes were examined at bregma locations of 0.97, − 2.03 (site of injection), and − 3.51 mm. Boxed diagrams on the right show different brain regions neuroanatomically connected to the dorsal hippocampus via either anterograde or retrograde pathways (left pointing arrows) or both pathways (double-headed arrows) (**b**, **d**, **f**). Brain regions showing AT8 immunoreactivity are indicated by bold and underlined text in the boxes (**b**, **d**, **f**). See also Additional file 1: Fig. S1. n = 3–4 mice/group

We next investigated whether APOE genotypes resulted in differential patterns of K18-tau aggregate induced ptau pathology originating from hippocampus and spreading along neuroanatomically connected areas (Fig. 2). We examined AT8 immunoreactivity at the site of injection (-2.03 mm from bregma) and two sites along the antero-posterior axis of K18-tau aggregate seeded PS/E2H, PS/E3H and PS/E4H mice (Fig. 2a, c, e). Based on our previous observations in K18-tau seeded PS19 mice [22], we selected brain areas that are directly connected to dorsal hippocampus via anterograde and retrograde pathways (Fig. 2b, d, f). At the site of injection, PS/E3H mice accumulated equivalent burden of AT8 tauopathy in both ipsilateral and contralateral hippocampus, while the other APOE genotypes (E2H and E4H) showed lower levels of ptau pathology (Fig. 2a, c, e). All three genotypes accumulated AT8-tau inclusions in the anterogradely connected thalamus and anterior cingulate area, with PS/E3H mice showing more abundance (E3H > E2H > E4H) (Fig. 2a–f). Dorsal areas of PS/E3H mice such as caudate putamen, periventricular region, septal nucleus and piriform cortex showed higher AT8 reactivity relative to PS/E2H and PS/E4H mice (Fig. 2a–f). We also observed differential transmission of tau into retrogradely connected regions, with PS/E3H mice showing AT8 immunoreactivity in all areas examined (Fig. 2b, d, f). While all three genotypes had tau pathology in entorhinal cortex and ventral dentate gyrus (E3H ~ E2H > E4H), areas like ventral tegmental area and raphe nucleus showed no significant tau pathology in PS/E2H and PS/E4H mice. The prefrontal cortex (ptau negative for PS/E4H) and mammillary areas (ptau negative for PS/E2H and PS/E4H) also showed differential tau immunoreactivity between the three genotypes. Overall, we found that the induction of ptau pathology by misfolded tau seeds was more widespread in the PS/E3H mice.

### Focused transcriptome analysis of K18-tau seeded PS19 mice homozygous for human APOE

**Fig. 3:**
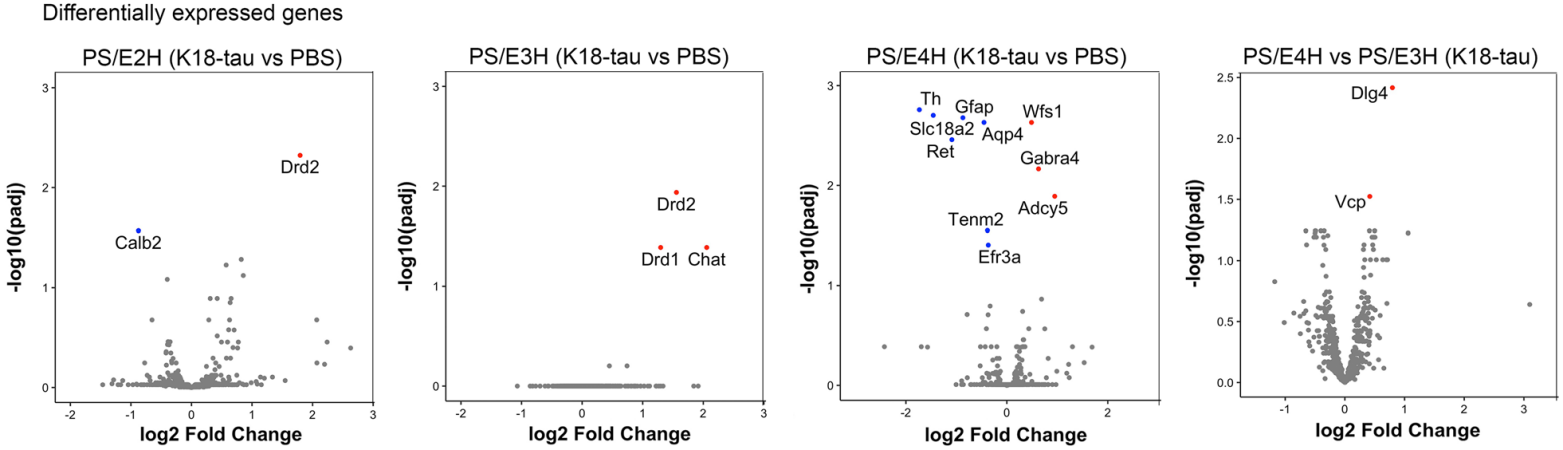
Differential gene expression patterns in K18-tau aggregate seeded PS19 mice homozygous for APOE. nCounter Neuropathology panel was used to assess differential gene expression patterns in K18-tau aggregate seeded PS/E2H, PS/E3H and PS/E4H mice relative to age- and genotype-matched PBS injected mice (left three panels). Genes differentially regulated in K18-tau aggregate seeded PS/E4H mice relative to PS/E3H mice is shown on the right panel. *p* values adjusted for multiple testing; FDR = 0.05. n = 3–4 mice/group

We used the 770-gene NanoString nCounter AD Neuropathology platform to identify induction of AD-related gene signatures in K18-tau aggregate seeded PS19xAPOE mice relative to PBS-injected genotype-matched mice (Fig. 3). PS/E3H and PS/E2H showed limited differential gene expression (DEG) patterns, with upregulation of Dopamine Receptor D2 (Drd2) gene as a common gene (Fig. 3). PS/E3H also showed upregulation of Drd1 and choline o-acetyl transferase (ChAT) genes. Surprisingly, the most changes in DEG were observed in PS/E4H mice (Fig. 3), even though these mice accumulated significantly lower AT8 burden relative to PS/E3H mice. The genes that were downregulated in PS/E4H mice included Aqp4, Gfap, Th, Ret/GDNF receptor, Slc18a2, Efr3a and Tenm2 whereas Wfs1, Adcy5 and Gabra4 were upregulated in K18-seeded PS/E4H mice relative to PBS-injected PS/E4H mice (Fig. 3). When K18-tau seeded PS/E4H mice were compared to K18-tau seeded PS/E3H mice, we found Valosin containing protein (Vcp) and Dlg4/Psd95 to be upregulated (Fig. 3). Overall, we observed modest changes in DEG profiles in K18-tau seeded PS19 mice and most DEG changes were associated with APOE4 allele.

### Gliosis profile in K18-tau aggregate seeded PS19 mice homozygous for human APOE

**Fig. 4:**
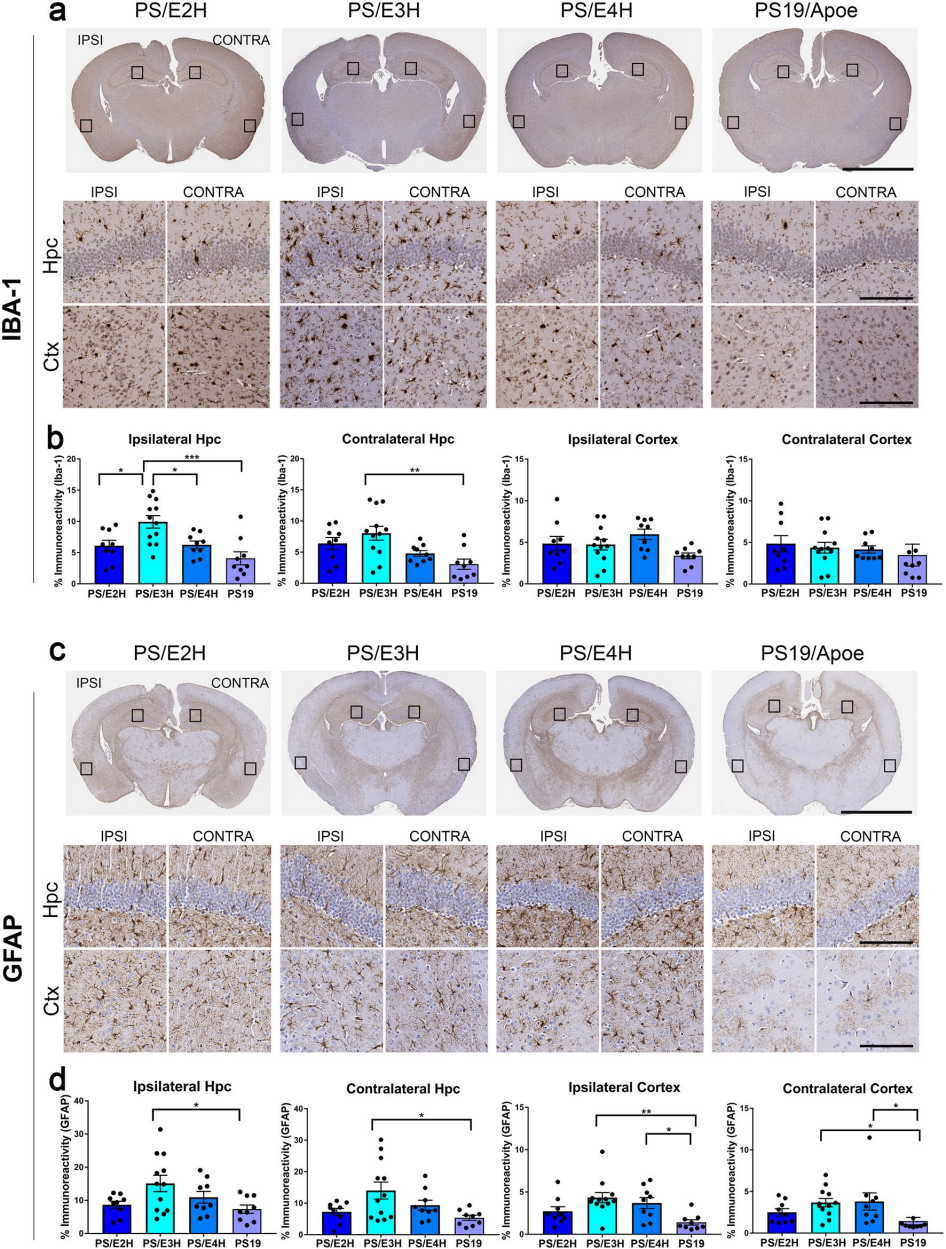
Induction of gliosis in K18-tau seeded PS19 mice homozygous for APOE. K18-tau fibrils were injected into the left hippocampus of 2.5-month-old mice and aged for 5 months. 7.5-month-old mice were analyzed for Iba-1 immunoreactive microgliosis (**a**, **b**) and GFAP-immunoreactive astrogliosis (**c**, **d**). Representative images from the hippocampus and cortex of K18-tau aggregate injected hemisphere (ipsilateral, ‘IPSI’) and uninjected hemisphere (contralateral, ‘CONTRA’) are shown (**a**, **c**). Quantification of the Iba-1 and GFAP immunostaining in 7.5-month-old mice is presented as % immunoreactivity in the cortex (Ctx) or hippocampus (Hpc) (**b**, **d**). Boxes in whole brain panel indicate selected areas used for high power zoomed panels. n = 9–12 mice/group. 1-way ANOVA *p < 0.05, **p < 0.01, ***p < 0.001. Scale bar: 3 mm (whole brain); 100 µm (hpc and ctx)

We analyzed how the induction of K18-seeded tauopathy influenced the proliferation of microglia and astrocytes in PS19 mice homozygous for APOE (Fig. 4; Additional file 4: Fig. S4e-h). Using antibodies against the microglial marker Iba-1 and astrocyte marker GFAP, we first established that the pattern of hippocampal microgliosis and astrogliosis was similar between the three APOE genotypes in the PBS-injected vehicle control group (Additional file 4: Fig. S4e-h). Interestingly, parental PS19 mice (with murine Apoe) injected with PBS showed lower astrocyte burden in the contralateral hippocampus and cortex, especially relative to PS/E4H mice (*p* < 0.01 in contralateral hippocampus and contralateral cortex) (Additional file 4: Fig. S4g-h). In the K18-seeded cohorts, we observed robust Iba-1 reactive microgliosis in the ipsilateral hippocampus of PS/E3H mice relative to all other genotypes (*p* < 0.05 to 0.001) (Fig. 4a, b). In the contralateral hippocampus, Iba-1 immunoreactivity in PS/E3H mice showed significant upregulation relative to PS19 mice (*p* < 0.01) and trended higher against PS/E4H mice (*p* = 0.071) (Fig. 4a, b). We also investigated the homeostatic microglia marker, Tmem119, and activated macrophage marker, Cd68, and did not find any APOE isoform-dependent upregulation in the K18-tau seeded PS19xAPOE homozygous cohorts (data not shown).

In K18-tau seeded mice, hippocampal astrogliosis did not show a clear APOE isoform-dependent effect (Fig. 4c, d). GFAP was only modestly upregulated in ipsilateral cortex (*p* < 0.05) of PS/E3H mice relative to PS/E4H mice (Fig. 4c, d). We observed the most change in PS/E3H mice compared to PS19 mice with murine Apoe. Astrogliosis was upregulated in the ipsilateral hippocampus (*p* < 0.05), contralateral hippocampus (*p* < 0.05), ipsilateral cortex (*p* < 0.01) and contralateral cortex (*p* < 0.05) of K18-tau aggregate seeded PS/E3H mice relative to parental PS19 mice (Fig. 4c, d).

### Gliosis profile in K18-tau aggregate seeded PS19 mice heterozygous for human APOE

We tested the gliosis profiles of PS19 mice that are heterozygous for APOE allele from the B6N2 and B6N1 generations. In the B6N2 generation, PBS injection did not cause any APOE-isoform dependent changes in microgliosis (Iba-1: Additional file 10: Fig. S10a-b) or astrogliosis (GFAP: Additional file 10: Fig. S10e-f). Following K18-tau aggregate seeding, we did not observe any APOE allele-dependent changes in microgliosis (Additional file 10: Fig. S10c-d). GFAP levels were upregulated only in the contralateral hippocampus of PS/E3h mice relative to PS/E2H mice (*p* < 0.05) while the changes in other areas only showed modest trends (Additional file 10: Fig. S10g-h).

In the B6N1 generation of seeded PS19xAPOE heterozygous mice, there were few differences in Iba-1 or GFAP reactivity among the APOE genotypes (Additional file 11: Fig. S11). PBS injection in these mice showed no significant APOE-associated changes in glial burden (Additional file 11: Fig. S11a, b, e, f). No appreciable changes were observed in microgliosis following K18-tau aggregate seeding in these mice (Additional file 11: Fig. S11c-d), consistent with observations from heterozygous mice from the B6N2 generation. K18-tau seeded PS/E2h mice showed increased astrogliosis in the ipsilateral (*p* < 0.01) and contralateral (*p* < 0.01) hippocampus compared to PS/E4h mice (Additional file 11: Fig. S11g-h). Together, these findings indicate that heterozygosity for human APOE alleles did not greatly influence microglial or astrocytic reaction to K18-tau seeds.

### Comparative analysis of human APOE and mouse Apoe in hippocampal K18-tau aggregate seeded mice

**Fig. 5:**
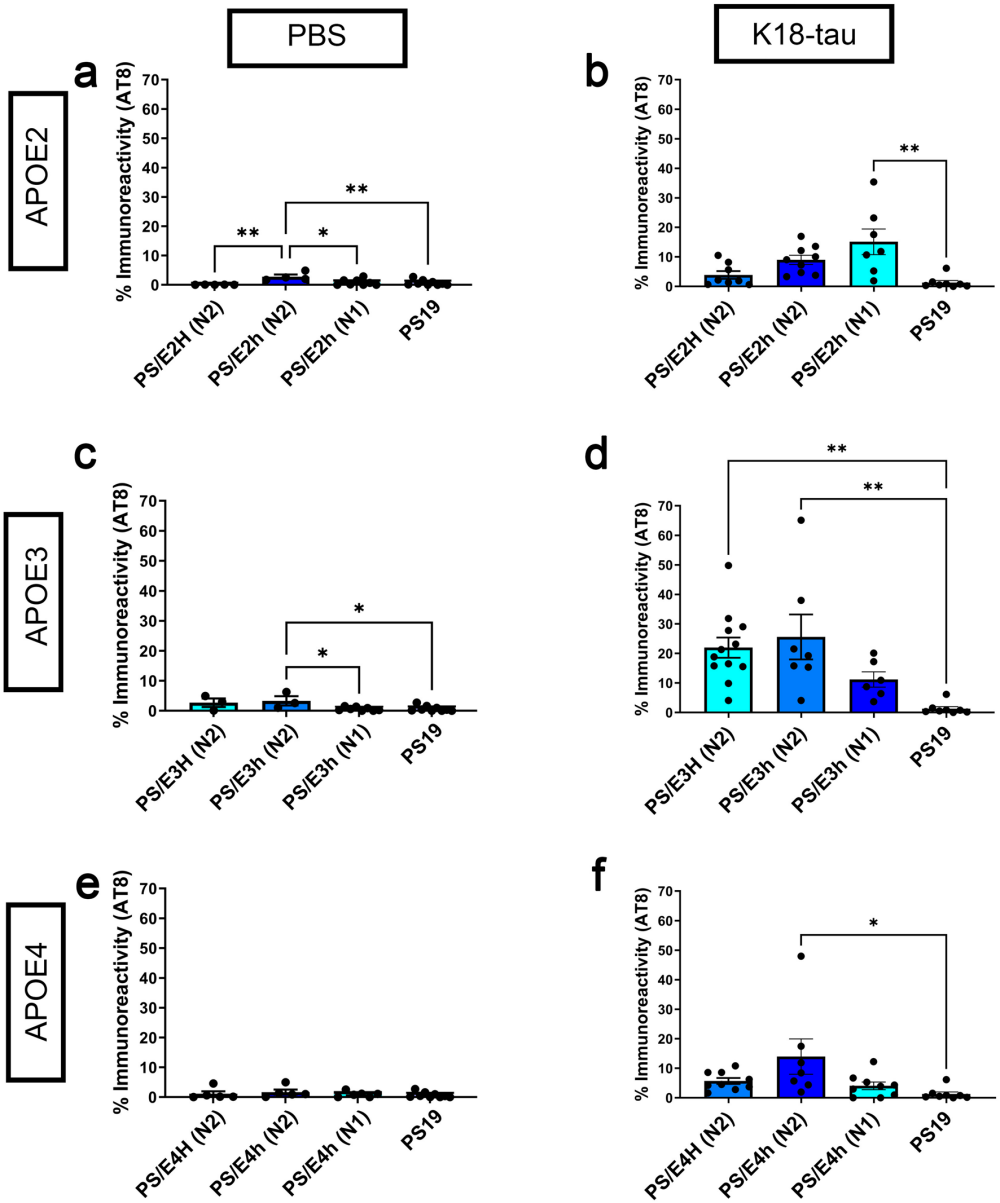
Comparative analysis of intrahippocampal pathology in PS19 mice carrying human APOE alleles or murine Apoe. Graphical representation of AT8 immunoreactivity levels in bigenic PS19 mice carrying human APOE alleles from the B6N2 and B6N1 generations and parental PS19 mice carrying mouse Apoe allele. N1 (B6N1) and N2 (B6N2) refer to two subsequent generations with slightly different mouse genetic backgrounds. AT8 burden from PBS injected mice (**a**, **c**, **e**) and K18-tau aggregate seeded mice (**b**, **d**, **f**) shown from three different PS19xAPOE cohorts. Data originally shown in Fig. 2, S8 and S9

Based on our AT8 immunostaining data described above from K18-tau aggregate seeded B6N1 and B6N2 generation mice, we re-evaluated tauopathy induction within individual APOE genotypes compared to mouse Apoe to assess the effects of background strain (Fig. 5a–f). In both the PBS-injected and K18-tau aggregate injected cohorts, we found APOE3 to be associated with higher AT8 immunostaining. While we did observe that increasing the contribution of C57BL/6 J background genes was associated with increased AT8 staining, the APOE genotype was clearly the stronger influence on ptau induction (Fig. 5d). In the K18-tau injected cohorts, we found that both APOE3 homozygous (*p* < 0.01) and APOE3 heterozygous (*p* < 0.01) mice from the B6N2 generation showed roughly equivalent AT8 burden which was significantly higher compared to parental PS19 mice (Fig. 5). For APOE4 and APOE2, we did not observe this effect (Fig. 5b, f). Indeed, homozygotes for both APOE4 and APOE2 showed lower pathogenic burden of tau relative to heterozygotes, while heterozygotes for APOE4 and APOE2 showed higher ptau compared to parental PS19 mice (Fig. 5b, f).

## Discussion

In this study, we report the comparative role of different human APOE isoforms and mouse Apoe in initiating tau seeding and spread along neuroanatomically connected brain regions. To our knowledge, this is the first study that examines the relative efficiency of APOE isoform-dependent tau seeding and early disease staging in a rodent model of Aβ-independent tauopathy. We observed that hippocampal induction and neuroanatomic transmission of ptau is exacerbated in presence of human APOE3 alleles. Indeed, both PS/E3H mice and its heterozygotic sibling PS/E3h mice (carrying one human APOE3 allele and one mouse Apoe allele) show increased ptau accumulation following hippocampal seeding with recombinant tau prions. Together, these data suggest that that the presence of human APOE3 significantly potentiates tau phosphorylation induced by exposure to recombinant K18-tau prions.

Previous studies have suggested that presence of APOE4 may have a pathogenic influence on tau pathology relative to APOE2 or APOE3. The first study to use bigenic PS19 tau mice and APOE mice reported that APOE4 triggers tau-mediated neurodegeneration, and subsequent work suggested that glial APOE4 underlies this pathological outcome [15–17]. In contrast to these studies, we primarily focused on ipsilateral and contralateral hemispheric ptau induction patterns after seeding. We did not observe obvious evidence of ventricle enlargement and neurodegeneration in our model as previously reported by Shi et al. [15], which could be due to the fact that the mice in our study were analyzed before they reached end-stage pathology. The differences in phenotypes may also be due to experimental methodology (tau seeding versus aging paradigm) or due to genotype-environment interactions, such as (mouse facility conditions or subtle variations in mouse background strain as has been noted by other studies [31].

In human patients, a handful of studies have reported direct association between regional tau deposition and APOE4 genotype [32]. Most studies suggest that the link between APOE4 and tangle pathology is either an indirect interaction or dependent on Aβ [6, 7, 33, 34]. In pure tauopathies, such as progressive supranuclear palsy (PSP) and argyrophilic grain disease (AGD), presence of APOE4 is also not correlated to tau burden [35–38]. A recent study, however, identified APOE2 as a risk factor in PSP supported by neuropathology data from mouse overexpression models [10]. Data from our mouse study, however, is more consistent with findings in human primary age-related tauopathy (PART). PART is defined as a spectrum of Aβ-negative tauopathy characterized by AD-type tangles in the hippocampus of elderly patients with mostly intact cognitive abilities [9]. These individuals also rarely display co-pathologies, such as α-synuclein or TDP-43 inclusions. In individuals with definite PART diagnosis, the allele frequency of APOE4 is 8–10% as opposed to ~ 60% for definite AD [39]. Thus, there is no significant association between APOE4 genotype and tau tangle burden in PART [40]. On the other hand, APOE2/APOE3 and APOE3/APOE3 individuals seem to display preferentially higher burden of tau pathology in these PART cohorts [41]. Thus, our hippocampal seeding paradigm in PS/E3H mice could be considered as recapitulating age-related tauopathy in the absence of co-occurring pathologies common in AD, such as Aβ, α-synuclein and TDP-43. Whether the unique observations in our study regarding the role of APOE3 in prionoid seeding of tau experimentally model aging related tauopathys observed in human PART requires further study.

The mechanism underlying the link between APOE3 and tau pathology seems uncertain, given that their primary sites of cellular origin are different. Tau is expressed and maintained intra-neuronally, while APOE is produced by glial cells and secreted where it often associates with parenchymal Aβ deposits. While tau can be secreted as discrete entities in AD [42] and also can be found bound to cored Aβ deposits, there is scant evidence of direct interaction between the two. Direct physical interaction between APOE and tau remains controversial, though some reports suggest that tau binds APOE in an isoform specific manner in vitro. For example, APOE3 (but not APOE4) preferentially bound recombinant 2/4R tau in vitro [43, 44]. Another report showed that non-lipidated APOE2 (but not APOE3 or APOE4) as well as astrocyte-secreted APOE2 or APOE3 (but not APOE4) lipoprotein particles preferentially bound to 2N/4R tau in vitro [10]. Overall, this would suggest that if APOE and tau were to interact with each other, both APOE2 and APOE3 could potentially affect tau metabolism and half-life. Whether the outcome of this potential interaction would be determined by the differential levels of secreted APOE observed among the different isoforms [45] or would be determined by the stability of the respective lipoprotein particles characteristic of the predominant APOE isoform present [46] will need cautious examination. Overall, these biochemical interaction data, albeit derived from recombinant constructs in an in vitro setting, are consistent with our observation that APOE3 seems to facilitate seeding-mediated accumulation of ptau.

In our study, we observed that the predominant effect of K18-tau seeding in the APOE3 mice was on ptau levels, and not necessarily NFT. This brings forth the idea that APOE3 could be influencing cellular kinases or phosphatases that influence tau phosphorylation. There is some intriguing data derived from human biomaterials that show APOE activates the ERK1/2 MAP kinases via dual leucine-zipper kinase (DLK) [47]. Another report investigated proteomic profile of young APOE4 carriers where several kinases related to tau phosphorylation, such as atypical protein kinase C (aPKC) PKC-ι, mitogen-activated protein kinase 12 (MAPK12), a member of the p38 MAPK family, Src family tyrosine kinases FYN, and Ca2+/calmodulin (CaM)-dependent protein kinase II (CaMKII) were identified [48]. Taken together, a gap in our knowledge remains whether APOE is functionally related to cellular metabolism that influences tau phosphorylation or facilitates intercellular transfer of tau or both in AD and related dementias.

Rodent modeling studies have unequivocally demonstrated the selective neurotoxicity of APOE4 through multiple mechanisms. [49, 50]. Expression of human APOE isoforms have differential effects on cognitive function, neuroplasticity, gliosis, neurodegeneration, Aβ pathology and tauopathy in preclinical mouse models—mice expressing APOE4 are generally more vulnerable compared to APOE2 or APOE3 expressing mice [49–51]. Notably, mouse Apoe is somewhat similar to human APOE4 [52–55], as knocking out mouse Apoe alleles reduces Aβ plaque deposition in APP mice [55] and reduces tau-induced neurodegeneration in PS19 mice [15], mirroring the pathogenic effect of human APOE4 in AD. In our study, however, we noted that all of the human APOE isoforms (most reliably APOE3) perform better in terms of prion-type seeding compared to mouse Apoe. This warrants careful consideration of tau neuropathology outcomes conducted in the presence of murine homologs of AD risk genes.

Although limited in scope because of its obvious bias in listing genes related exclusively to AD neuropathology, the NanoString DEG nonetheless indicates certain interesting aspects. Firstly, we noticed that the extent of significant DEG was more profound in PS/E4H mice and rather limited in PS/E3H mice, relative to their genotype- and age-matched PBS-seeded cohorts. PS/E3H showed upregulation of Drd1, Drd2 and ChAT genes, implicating tau-APOE3 function in Parkinsonian dysfunction [28]. This is consistent with many of PD-associated areas, such as ventral tegmental area, hypothalamus and locus ceruleus accumulating robust tau pathology in our PS/E3H cohort. On the other hand, PS/E4H mice showed gene expression changes that were related to canonical astrocytic signaling (Aqp4 and Gfap), Ca^2+^ signaling (Wfs1, Adcy5 and Ret) and dopaminergic neuronal homeostasis (Th, Ret or GDNF receptor and Slc18a2). Reduced Tenm2, as noted in K18-tau seeded PS/E4H mice, has been associated with atrophy patterns in genetic FTD [56]. Secondly, comparing the gene expression profile of the PS/E4H mice with PS/E3H mice revealed that K18-tau seeding induces Vcp gene expression in PS/E4H mice, in spite of these mice accumulating lower tau pathology than PS/E3H mice. Vcp is significant in this respect as mutations in Vcp cause a specific form of FTD with inclusion body myositis and Paget’s bone disease [57], which is itself associated with APOE4 genotype [58]. Given this established association between VCP and APOE4 [11], this would suggest that APOE4 individuals could be at a higher risk of pathogenic outcomes, even with lower burden of regional tau deposition. It is tempting to suggest that this data implies that APOE3 has a protective role in individuals who accumulate age-related tau but do not undergo neurocognitive decline. Future studies, including behavioral tests to measure learning and memory dysfunction, could provide insights into the interaction of aging factors, tau seeding-induced pathology and FTD-related risk factors in APOE4 mice relative to APOE3 mice.

While our study provides a systematic neuropathological analysis in tau mice carrying different APOE isoforms, there are several open-ended questions raised by our current observations. One question that emerged is the extent that background strain influences seeding and transmission of tau in rodent models. In the B6N1 generation of crosses when all mice were heterozygous for the APOE allele, there was no consistent statistical difference in AT8 ptau burden across genotypes (see Fig. 5). In the B6N2 generation, we began to see hints that mice with one APOE3 allele have more tau pathology (see Fig. 5). Only in the mice that were homozygous for each APOE allele did we consistently observe PS/E3H mice to exhibit higher levels of AT8 and PHF-1 reactive pathology as compared to all other genotypes (see Figs. 1, 5). Interestingly, however, our comparison of tau burden within genotype demonstrated that in the B6N2 generation, homozygous PS/E3H and heterozygous PS/E3h exhibit similar levels of AT8 positivity, suggesting that a single APOE3 allele may be sufficient to influence tau phosphorylation.

A limitation of our model is using heparin-induced K18-tau filaments which do not necessarily recapitulate the human brain-associated structural polymorphs [59, 60]. We used this form of recombinant tau prion as K18-tau fibrils have been shown to misfold into PHFs and have been functionally validated by research groups as having self-templating properties [19, 22, 61]. Unexpectedly, the PS19xAPOE mice injected with K18-tau fibrils did not induce argentophilic or MC1-reactive tau pathology. The primary presentation of altered tau in all three human APOE genotypes was increased levels of ptau. It is possible that tau seeds derived from human AD brain will produce different outcomes and these could be investigated in the future. Another aspect would be to validate the observed larger effect of APOE4 on gene expression changes in NanoString panel by using unbiased techniques, such as RNAseq.

In conclusion, we show that presence of APOE3 exacerbates the phosphorylation of tau that has been induced to misfold by exogenous tau prions. Additional experimentation using human brain derived tau seeds would allow us to further confirm the association of APOE3 with pathology progression markers in primary and secondary tauopathies.

## Supplementary Information

**Additional file 1. Figure S1:** Representative images of ptau pathologies corresponding to neuropathology scores. Immunohistochemical images of AT8 staining were scored using a neuropathology score sheet by 2 blinded observers. These scores were used to impute ptau patterns as depicted in Fig. 2. Representative images depict the burden of ptau neuropathology corresponding to score of 0 (no pathology), 1 (low pathology), 2 (medium pathology) and 3 (high pathology) (a). The key to the heat map scores is provided (a). Representative AT8 staining from different brain regions of PS19xAPOE mice shown to illustrate the effects of tau seeding in different brain regions used to generate ptau burden data in Figure 2. Scale Bar 100 µm (a); 70 µm (b).

**Additional file 2. Figure S2:** Tau levels in PS19xAPOE colony. Immunoblotting for human tau (CP27 antibody) in APOE TR mice, PS19 mice, PS19 mice heterozygous (HET) for APOE (B6N2 generation) and PS19 mice homozygous (HOM) for APOE (B6N2 generation). GAPDH marks the housekeeping control for the immunoblots (a). Quantification of tau protein levels (normalized to GAPDH) is shown (b). N=3 mice/group. Representative immunohistochemistry using an APOE4 specific antibody on PS19 mice homozygous for APOE (PS/E2H, PS/E3H and PS/E4H mice, c) and PS19 mice heterozygous for APOE (PS/E2h, PS/E3h, PS/E4h, d) shown. Presentative images from hippocampus (Hpc) and cortex (Ctx) shown. n=3 mice from each colony (representing different founders). Scale: 3 mm (whole brain); 100 µm (zoomed panels).

**Additional file 3. Figure S3:** APOE levels in PS19xAPOE homozygous mice. PS19 mice homozygous for APOE alleles were analyzed for APOE alleles using immunoblotting (a), immunohistochemistry (b-c) and co-immunofluorescence (d). a. Representative immunoblot and quantitation of APOE (standardized to housekeeping gene GAPDH) from RIPA-soluble forebrain lysates of PS19 mice, homozygous APOE TR mice and bigenic PS19 mice homozygous for APOE alleles. 1-way Anova, **p<0.01, *p<0.05. n=3 mice/genotype. Since the APOE antibody is specific for human/primate APOE, mouse Apoe shows lower signal and thus was excluded from analysis. b-c. APOE immunohistochemistry on spinal cord and brains of bigenic PS19 mice homozygous for APOE alleles. Gr Matter: grey matter; Wh Matter: white matter. Scale bar: Spinal cord: 150 µm (left panel), 50 µm (right panel); Brain: 500 µm (main panel), 50 µm (inset). n=3 mice/genotype. d. Co-immunofluorescence showing presence of APOE in astrocytes (GFAP immunostaining) and microglia (Iba-1 immunostaining) in brains of bigenic PS19 mice homozygous for APOE alleles. Arrows indicate co-localized immunofluorescence signals. Scale bar: 50 µm. n=3 mice/genotype.

**Additional file 4. Figure S4:** Neuropathological characterization of PBS-injected PS19 mice homozygous for APOE. PBS was injected into the left hippocampus of 2.5-month-old PS/E2H, PS/E3H, PS/E4H mice (B6N2 generation) and PS19 mice. Representative images from the hippocampus (Hpc) and cortex (Ctx) of injected (ipsilateral, ‘IPSI’) and uninjected (contralateral, ‘CONTRA’) hemispheres showing pathology in 7.5 month old PS/E2H, PS/E3H and PS/E4H mice are shown. Tau pathology is assessed using AT8 and PHF1 antibodies (a, c), microgliosis using Iba-1 antibody (e) and astrogliosis using GFAP antibody (g). Quantification of immunostaining is presented as % immunoreactivity in the cortex (Ctx) or hippocampus (Hpc) of ipsilateral and contralateral hemispheres (b, d, f, h). n=3-5 mice/group. 1-way ANOVA *p<0.05, **p<0.01. Scale bar: 70 µm.

**Additional file 5. Figure S5:** Phosphorylated tau pathology in spinal cords of K18-tau aggregate injected PS19 mice homozygous for APOE. K18-tau aggregate or PBS was injected into the left hippocampus of 2.5-month-old PS/E2H, PS/E3H and PS/E4H mice (B6N2 generation). Representative images of AT8 immunostaining and quantitative analysis of AT8 burden from the spinal cords of injected PS19xOE mice are shown. n=3-5 mice (PBS injection group) and n= 8-12 mice/group (K18-tau injection group). 2-tailed t test. Scale bar: 500µm.

**Additional file 6. Figure S6:** ptau and NFT pathology in K18-tau aggregate injected PS19 mice homozygous for APOE. K18-tau aggregate was injected into the left hippocampus of 2.5-month-old PS/E2H, PS/E3H, PS/E4H mice (B6N2 generation) and PS19 mice. Representative images from the hippocampus (Hpc) and cortex (Ctx) of injected (ipsilateral, ‘IPSI’) and uninjected (contralateral, ‘CONTRA’) hemispheres showing pathology in PS19xPOE mice. Phosphorylated tau was assessed using PHF1 antibody (a, b). 1-way Anova; ***p<0.001, **p<0.01, *p<0.05. NFT pathology was assessed using Gallyas silver staining (c, d). Quantification is presented from cortex (Ctx) or hippocampus (Hpc) of ipsilateral and contralateral hemispheres are presented from PS/E2H, PS/E3H, PS/E4H and PS19 mice (e). n=8-12 mice/group. 2-tailed t test; *p<0.05. Scale bar: 100 µm (a), 100 µm (c-d, Hpc) 70 µm (c-d, Ctx).

**Additional file 7. Figure S7: ** Misfolded tau in K18-tau aggregate injected PS19 mice homozygous for APOE. K18-tau aggregate was injected into the left hippocampus of 2.5-month-old PS/E2H, PS/E3H and PS/E4H mice (B6N2 generation). Representative images from the hippocampus (Hpc) and cortex (Ctx) of injected (ipsilateral, ‘IPSI’) and uninjected (contralateral, ‘CONTRA’) hemispheres showing pathology in PS19xAPOE mice. Misfolded tau pathology is assessed using MC1 antibody count (a, b) and Tau C3 antibody reactivity (c, d). Quantification is presented from cortex (Ctx) or hippocampus (Hpc) of ipsilateral and contralateral hemispheres (b, d) underneath corresponding image panels. n=8-12 mice/group. 1-way ANOVA *p<0.05. Scale bar: 100 µm.

**Additional file 8. Figure S8:** ptau and misfolded tau in PS19 mice heterozygous for APOE (B6N2 generation) injected with K18-tau aggregates in the hippocampus. K18-tau was injected into the left hippocampus of 2.5-month-old PS/E2h, PS/E3h and PS/E4h mice (B6N2 generation) and aged for 5 months. Representative images from the hippocampus (Hpc) and cortex (Ctx) of injected (ipsilateral, ‘IPSI’) and uninjected (contralateral, ‘CONTRA’) hemispheres showing pathology in PS/E2h, PS/E3h and PS/E4h mice. ptau is assessed by AT8 (a, b) and PHF1 antibodies (c, d) and misfolded tau pathology is assessed MC1 antibody (e, f). Quantification of % immunoreactivity is presented from cortex (Ctx) or hippocampus (Hpc) of ipsilateral and contralateral hemispheres (b, d, f) underneath corresponding stained panels. n=7-10 mice/group. 1-way ANOVA *p<0.05, **p<0.01. Scale bar: 70 µm.

**Additional file 9. Figure S9:** pau levels in PS19 mice heterozygous for APOE (B6N1 generation) injected with K18-tau aggregates in the hippocampus. K18-tau aggregates or PBS was injected into the left hippocampus of 2.5-month-old PS/E2h, PS/E3h and PS/E4h mice (B6N1 generation) and aged for 5 months. Representative images from the hippocampus (Hpc) and cortex (Ctx) of injected (ipsilateral, ‘IPSI’) and uninjected (contralateral, ‘CONTRA’) hemispheres showing pathology in PS/E2h, PS/E3h and PS/E4h mice. ptau is assessed by AT8 (a, b, c, d) and PHF1 antibodies (e, f, g, h). Quantification of % immunoreactivity is presented from cortex (Ctx) or hippocampus (Hpc) of ipsilateral and contralateral hemispheres of K18-tau aggregate (c, d, g, h) or PBS injected (a, b, e, f) mice. n= 7-10 mice/genotype (K18-tau aggregate group); n= 6-8 mice/genotype (PBS group). 1-way ANOVA *p<0.05, **p<0.01. Scale bar: 70 µm.

**Additional file 10. Figure S10:** Gliosis in PS19 mice heterozygous for APOE (B6N2 generation) injected with K18-tau aggregates in the hippocampus. K18-tau aggregates or PBS was injected into the left hippocampus of 2.5-month-old PS/E2h, PS/E3h and PS/E4h mice (B6N2 generation) and aged for 5 months. Representative images from the hippocampus (Hpc) and cortex (Ctx) of injected (ipsilateral, ‘IPSI’) and uninjected (contralateral, ‘CONTRA’) hemispheres showing pathology in PS/E2h, PS/E3h and PS/E4h mice. Microgliosis was assessed using Iba-1 antibody (a-d) and astrogliosis was assessed using GFAP antibody (e-h). Quantification of % immunoreactivity is presented from cortex (Ctx) or hippocampus (Hpc) of ipsilateral and contralateral hemispheres of K18-tau aggregate (c, d, g, h) or PBS injected (a, b, e, f) mice. n= 7-10 mice/genotype (K18-tau aggregate group); n= 3-5 mice/genotype (PBS group). 1-way ANOVA *p<0.05, **p<0.01. Scale bar: 70 µm.

**Additional file 11. Figure S11:** Gliosis in PS19 mice heterozygous for APOE (B6N1 generation) injected with K18-tau aggregates in the hippocampus. K18-tau aggregates or PBS was injected into the left hippocampus of 2.5-month-old PS/E2h, PS/E3h and PS/E4h mice (B6N1 generation) and aged for 5 months. Representative images from the hippocampus (Hpc) and cortex (Ctx) of injected (ipsilateral, ‘IPSI’) and uninjected (contralateral, ‘CONTRA’) hemispheres showing pathology in PS/E2h, PS/E3h and PS/E4h mice. Microgliosis was assessed using Iba-1 antibody (a-d) and astrogliosis was assessed using GFAP antibody (e-h). Quantification of % immunoreactivity is presented from cortex (Ctx) or hippocampus (Hpc) of ipsilateral and contralateral hemispheres of K18-tau aggregate (c, d, g, h) or PBS injected (a, b, e, f) mice. n= 7-10 mice/genotype (K18-tau aggregate group); n= 6-8 mice/genotype (PBS group). 1-way ANOVA *p<0.05, **p<0.01. Scale bar: 70 µm.

**Additional file 12. Table S1:**. Description of sample numbers and sex distribution within study cohorts. **Table S2**. List of antibodies used in the study. **Table S3**. Table summarizing overall antibody and histological staining data. **Table S4**. P-values for IHC data obtained APOE homozygous (B6N2) mice injected with K18-tau or PBS. **Table S5**. P-values for IHC data obtained from APOE heterozygous (B6N2) mice injected with K18-tau or PBS. **Table S6**. P-values for IHC data obtained from APOE heterozygous (B6N1) mice injected with K18-tau or PBS. **Table S7**. P-values of AT8 IHC (spinal cord) from APOE homozygous (B6N2) mice injected with K18-tau or PBS.

## Data Availability

All data and code are available from the corresponding author upon reasonable request following publication.

## Abbreviations

Aβ: Amyloid β
AD: Alzheimer’s disease
AGD: Argyrophilic grain disease
aPKC: Atypical protein kinase C
APOE: Apolipoprotein E
APOE TR: Apolipoprotein E targeted replacement mouse
CaMKII: Ca2+/calmodulin (CaM)-dependent protein kinase II
ChAT: Choline o-acetyl transferase
Contra: Contralateral hemisphere
Ctx: Cortex
DAM: Damage associated microglia
DEG: Differential gene expression
DLK: Dual Leucine-zipper Kinase
Drd2: Dopamine Receptor D2
FTD: Frontotemporal dementia
Hpc: Hippocampus
IHC: Immunohistochemistry
Ipsi: Ipsilateral hemisphere
MAPK12: Mitogen-activated protein kinase 12
MGnD: Microglial neurodegenerative phenotype
PART: Primary age-related tauopathy
PSP: Progressive supranuclear palsy
ptau: Phosphorylated tau
Vcp: Valosin containing protein
