# Supplementary figures and images for: Impact of APOE genotype on prion-type propagation of tauopathy
Source: Acta Neuropathol Commun. 2022 Apr 19;10:57. doi: 10.1186/s40478-022-01359-y (PMC9019935; doi:10.1186/s40478-022-01359-y)

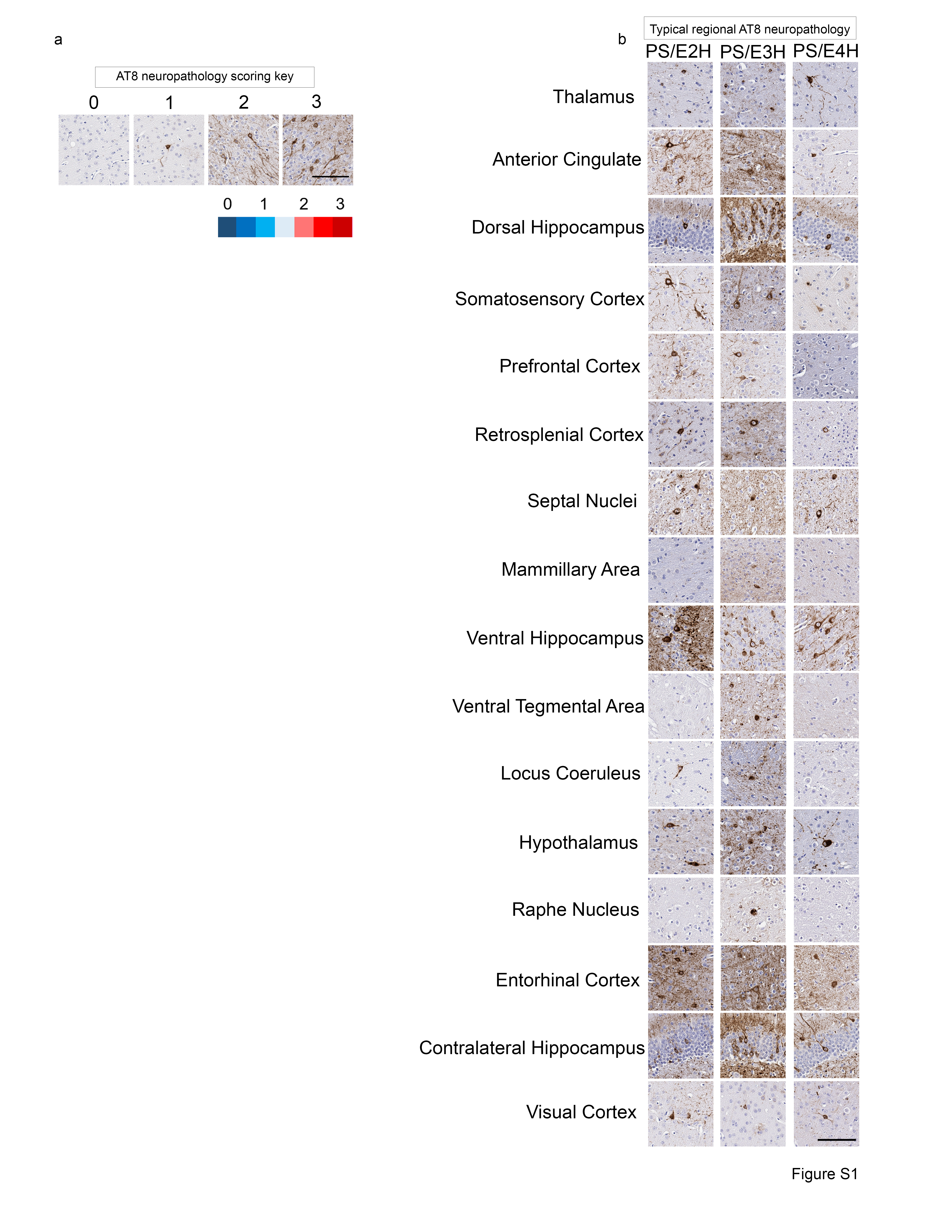

Supplement: Supplementary file 1 — Additional file 1. Figure S1: Representative images of ptau pathologies corresponding to neuropathology scores. Immunohistochemical images of AT8 staining were scored using a neuropathology score sheet by 2 blinded observers. These scores were used to impute ptau patterns as depicted in Fig. 2. Representative images depict the burden of ptau neuropathology corresponding to score of 0 (no pathology), 1 (low pathology), 2 (medium pathology) and 3 (high pathology) (a). The key to the heat map scores is provided (a). Representative AT8 staining from different brain regions of PS19xAPOE mice shown to illustrate the effects of tau seeding in different brain regions used to generate ptau burden data in Figure 2. Scale Bar 100 µm (a); 70 µm (b). [file 40478_2022_1359_MOESM1_ESM.jpg]

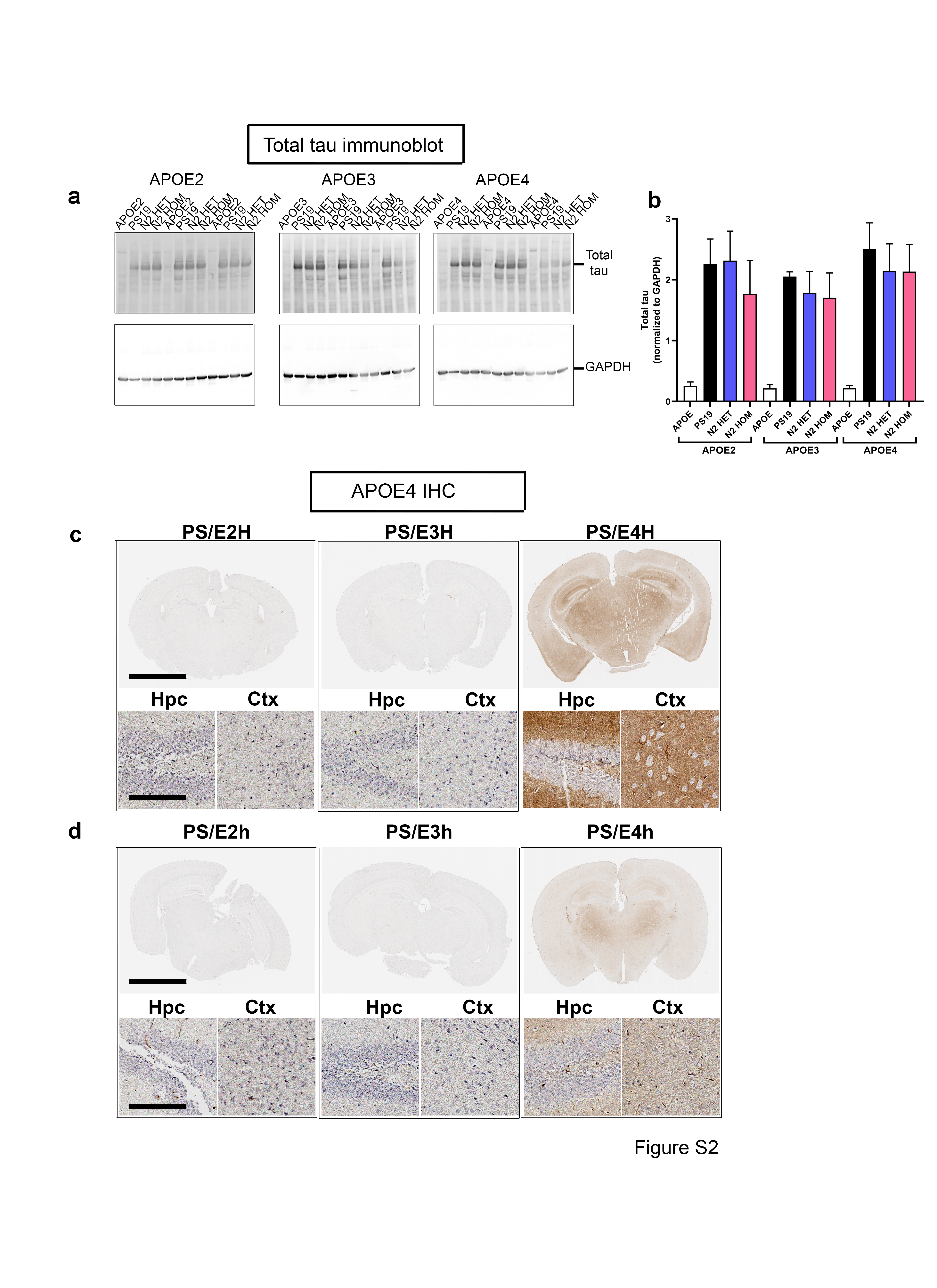

Supplement: Supplementary file 2 — Additional file 2. Figure S2: Tau levels in PS19xAPOE colony. Immunoblotting for human tau (CP27 antibody) in APOE TR mice, PS19 mice, PS19 mice heterozygous (HET) for APOE (B6N2 generation) and PS19 mice homozygous (HOM) for APOE (B6N2 generation). GAPDH marks the housekeeping control for the immunoblots (a). Quantification of tau protein levels (normalized to GAPDH) is shown (b). N=3 mice/group. Representative immunohistochemistry using an APOE4 specific antibody on PS19 mice homozygous for APOE (PS/E2H, PS/E3H and PS/E4H mice, c) and PS19 mice heterozygous for APOE (PS/E2h, PS/E3h, PS/E4h, d) shown. Presentative images from hippocampus (Hpc) and cortex (Ctx) shown. n=3 mice from each colony (representing different founders). Scale: 3 mm (whole brain); 100 µm (zoomed panels). [file 40478_2022_1359_MOESM2_ESM.jpg]

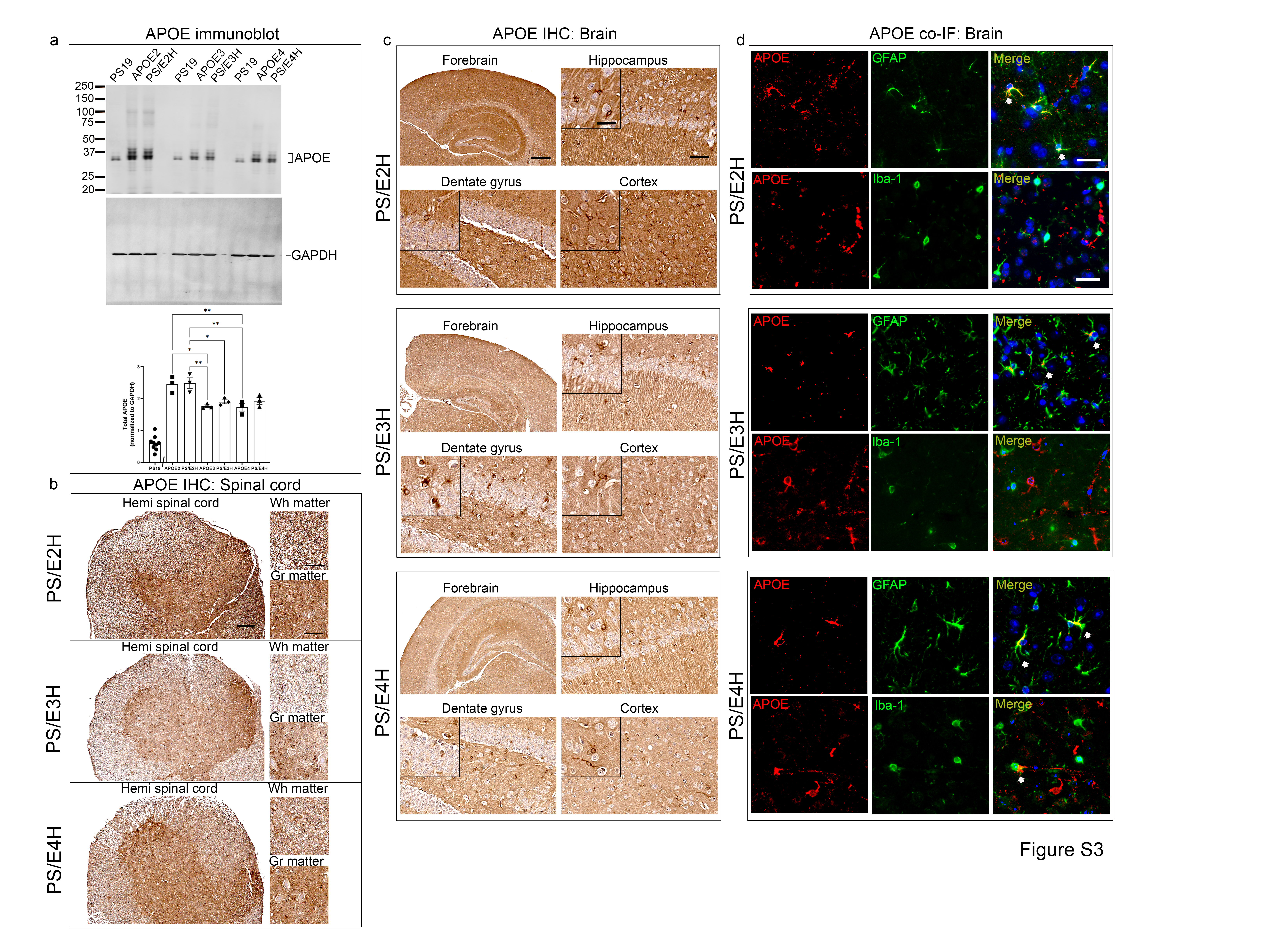

Supplement: Supplementary file 3 — Additional file 3. Figure S3: APOE levels in PS19xAPOE homozygous mice. PS19 mice homozygous for APOE alleles were analyzed for APOE alleles using immunoblotting (a), immunohistochemistry (b-c) and co-immunofluorescence (d). a. Representative immunoblot and quantitation of APOE (standardized to housekeeping gene GAPDH) from RIPA-soluble forebrain lysates of PS19 mice, homozygous APOE TR mice and bigenic PS19 mice homozygous for APOE alleles. 1-way Anova, **p<0.01, *p<0.05. n=3 mice/genotype. Since the APOE antibody is specific for human/primate APOE, mouse Apoe shows lower signal and thus was excluded from analysis. b-c. APOE immunohistochemistry on spinal cord and brains of bigenic PS19 mice homozygous for APOE alleles. Gr Matter: grey matter; Wh Matter: white matter. Scale bar: Spinal cord: 150 µm (left panel), 50 µm (right panel); Brain: 500 µm (main panel), 50 µm (inset). n=3 mice/genotype. d. Co-immunofluorescence showing presence of APOE in astrocytes (GFAP immunostaining) and microglia (Iba-1 immunostaining) in brains of bigenic PS19 mice homozygous for APOE alleles. Arrows indicate co-localized immunofluorescence signals. Scale bar: 50 µm. n=3 mice/genotype. [file 40478_2022_1359_MOESM3_ESM.jpg]

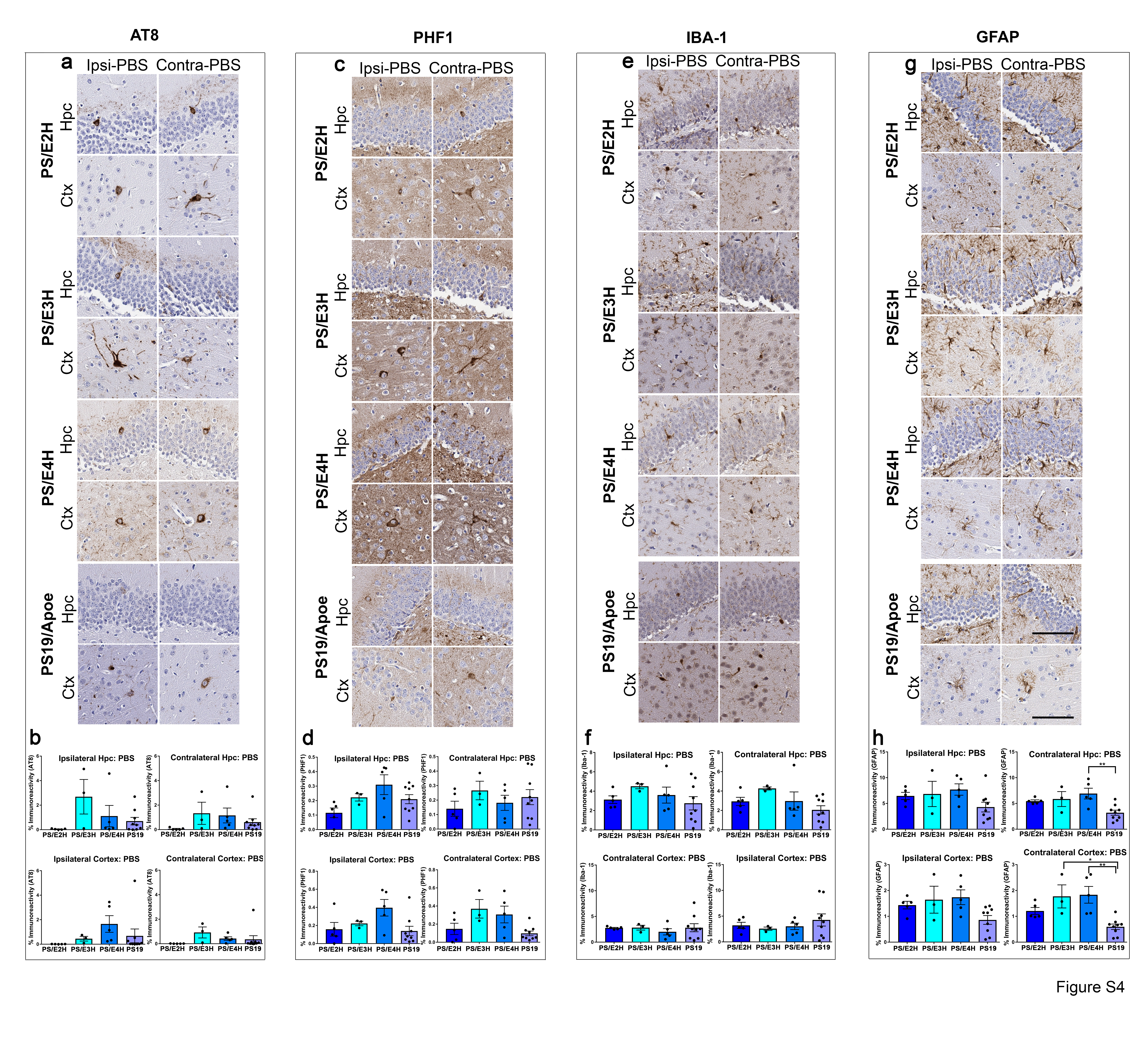

Supplement: Supplementary file 4 — Additional file 4. Figure S4: Neuropathological characterization of PBS-injected PS19 mice homozygous for APOE. PBS was injected into the left hippocampus of 2.5-month-old PS/E2H, PS/E3H, PS/E4H mice (B6N2 generation) and PS19 mice. Representative images from the hippocampus (Hpc) and cortex (Ctx) of injected (ipsilateral, ‘IPSI’) and uninjected (contralateral, ‘CONTRA’) hemispheres showing pathology in 7.5 month old PS/E2H, PS/E3H and PS/E4H mice are shown. Tau pathology is assessed using AT8 and PHF1 antibodies (a, c), microgliosis using Iba-1 antibody (e) and astrogliosis using GFAP antibody (g). Quantification of immunostaining is presented as % immunoreactivity in the cortex (Ctx) or hippocampus (Hpc) of ipsilateral and contralateral hemispheres (b, d, f, h). n=3-5 mice/group. 1-way ANOVA *p<0.05, **p<0.01. Scale bar: 70 µm. [file 40478_2022_1359_MOESM4_ESM.jpg]

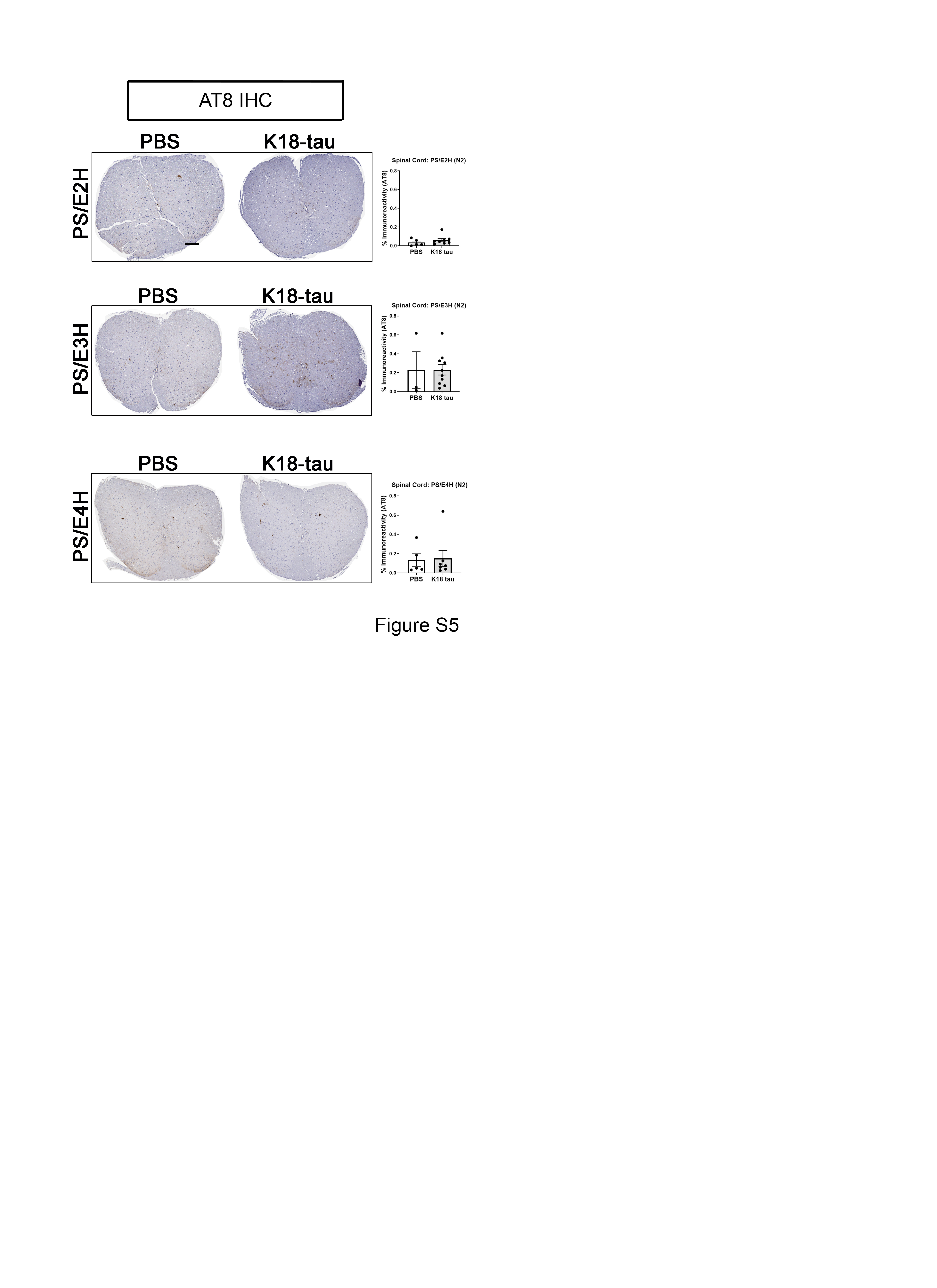

Supplement: Supplementary file 5 — Additional file 5. Figure S5: Phosphorylated tau pathology in spinal cords of K18-tau aggregate injected PS19 mice homozygous for APOE. K18-tau aggregate or PBS was injected into the left hippocampus of 2.5-month-old PS/E2H, PS/E3H and PS/E4H mice (B6N2 generation). Representative images of AT8 immunostaining and quantitative analysis of AT8 burden from the spinal cords of injected PS19xOE mice are shown. n=3-5 mice (PBS injection group) and n= 8-12 mice/group (K18-tau injection group). 2-tailed t test. Scale bar: 500µm. [file 40478_2022_1359_MOESM5_ESM.jpg]

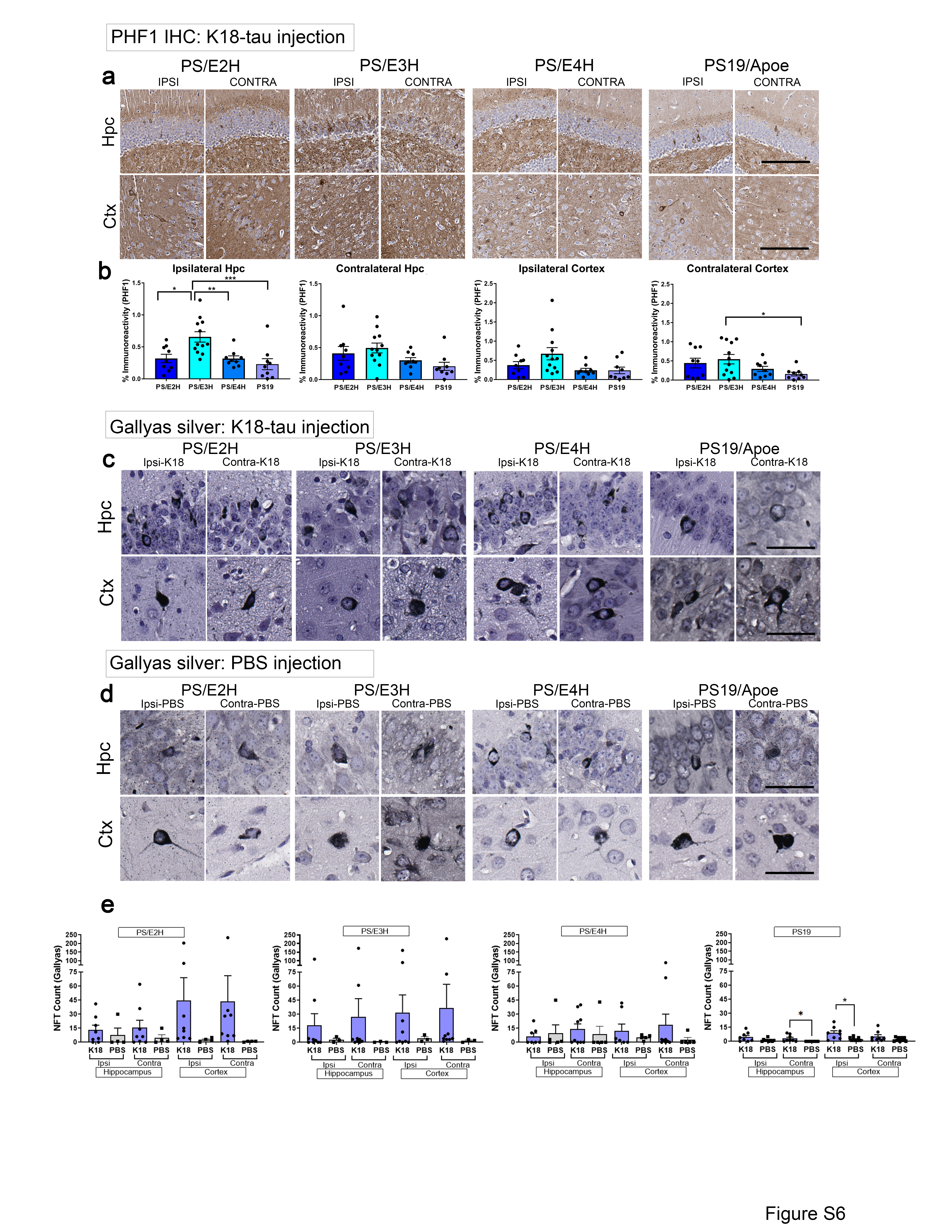

Supplement: Supplementary file 6 — Additional file 6. Figure S6: ptau and NFT pathology in K18-tau aggregate injected PS19 mice homozygous for APOE. K18-tau aggregate was injected into the left hippocampus of 2.5-month-old PS/E2H, PS/E3H, PS/E4H mice (B6N2 generation) and PS19 mice. Representative images from the hippocampus (Hpc) and cortex (Ctx) of injected (ipsilateral, ‘IPSI’) and uninjected (contralateral, ‘CONTRA’) hemispheres showing pathology in PS19xPOE mice. Phosphorylated tau was assessed using PHF1 antibody (a, b). 1-way Anova; ***p<0.001, **p<0.01, *p<0.05. NFT pathology was assessed using Gallyas silver staining (c, d). Quantification is presented from cortex (Ctx) or hippocampus (Hpc) of ipsilateral and contralateral hemispheres are presented from PS/E2H, PS/E3H, PS/E4H and PS19 mice (e). n=8-12 mice/group. 2-tailed t test; *p<0.05. Scale bar: 100 µm (a), 100 µm (c-d, Hpc) 70 µm (c-d, Ctx). [file 40478_2022_1359_MOESM6_ESM.jpg]

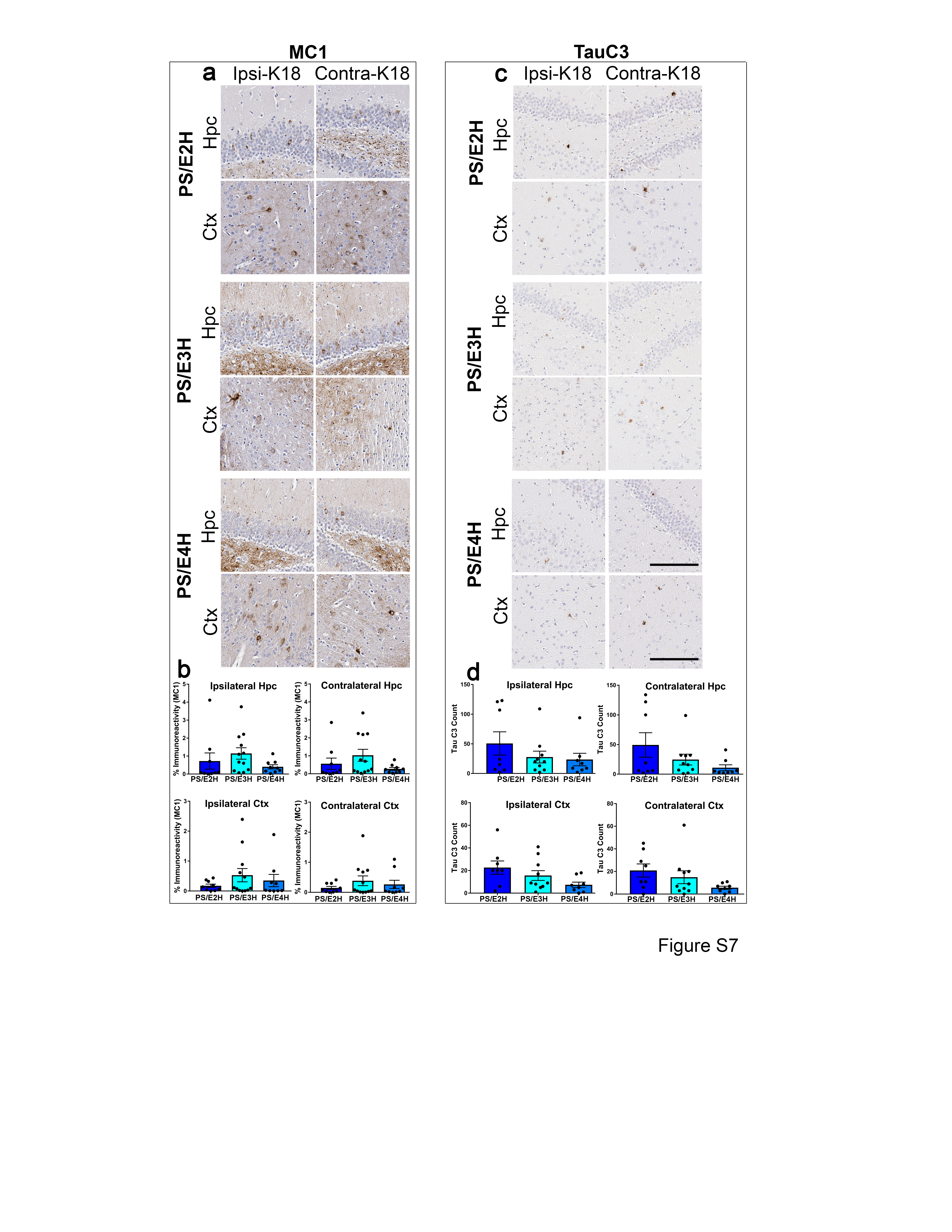

Supplement: Supplementary file 7 — Additional file 7. Figure S7: Misfolded tau in K18-tau aggregate injected PS19 mice homozygous for APOE. K18-tau aggregate was injected into the left hippocampus of 2.5-month-old PS/E2H, PS/E3H and PS/E4H mice (B6N2 generation). Representative images from the hippocampus (Hpc) and cortex (Ctx) of injected (ipsilateral, ‘IPSI’) and uninjected (contralateral, ‘CONTRA’) hemispheres showing pathology in PS19xAPOE mice. Misfolded tau pathology is assessed using MC1 antibody count (a, b) and Tau C3 antibody reactivity (c, d). Quantification is presented from cortex (Ctx) or hippocampus (Hpc) of ipsilateral and contralateral hemispheres (b, d) underneath corresponding image panels. n=8-12 mice/group. 1-way ANOVA *p<0.05. Scale bar: 100 µm. [file 40478_2022_1359_MOESM7_ESM.jpg]

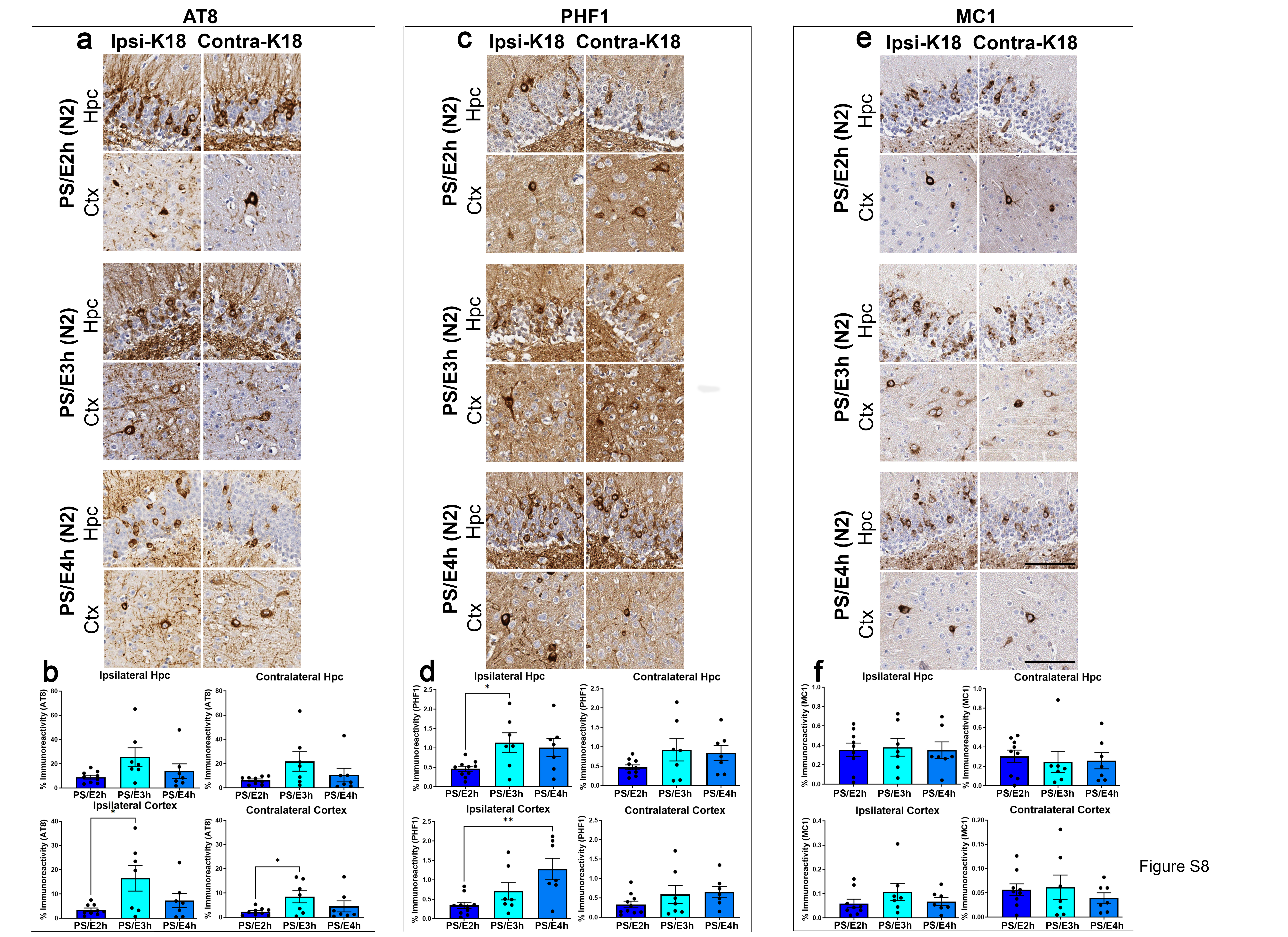

Supplement: Supplementary file 8 — Additional file 8. Figure S8: ptau and misfolded tau in PS19 mice heterozygous for APOE (B6N2 generation) injected with K18-tau aggregates in the hippocampus. K18-tau was injected into the left hippocampus of 2.5-month-old PS/E2h, PS/E3h and PS/E4h mice (B6N2 generation) and aged for 5 months. Representative images from the hippocampus (Hpc) and cortex (Ctx) of injected (ipsilateral, ‘IPSI’) and uninjected (contralateral, ‘CONTRA’) hemispheres showing pathology in PS/E2h, PS/E3h and PS/E4h mice. ptau is assessed by AT8 (a, b) and PHF1 antibodies (c, d) and misfolded tau pathology is assessed MC1 antibody (e, f). Quantification of % immunoreactivity is presented from cortex (Ctx) or hippocampus (Hpc) of ipsilateral and contralateral hemispheres (b, d, f) underneath corresponding stained panels. n=7-10 mice/group. 1-way ANOVA *p<0.05, **p<0.01. Scale bar: 70 µm. [file 40478_2022_1359_MOESM8_ESM.jpg]

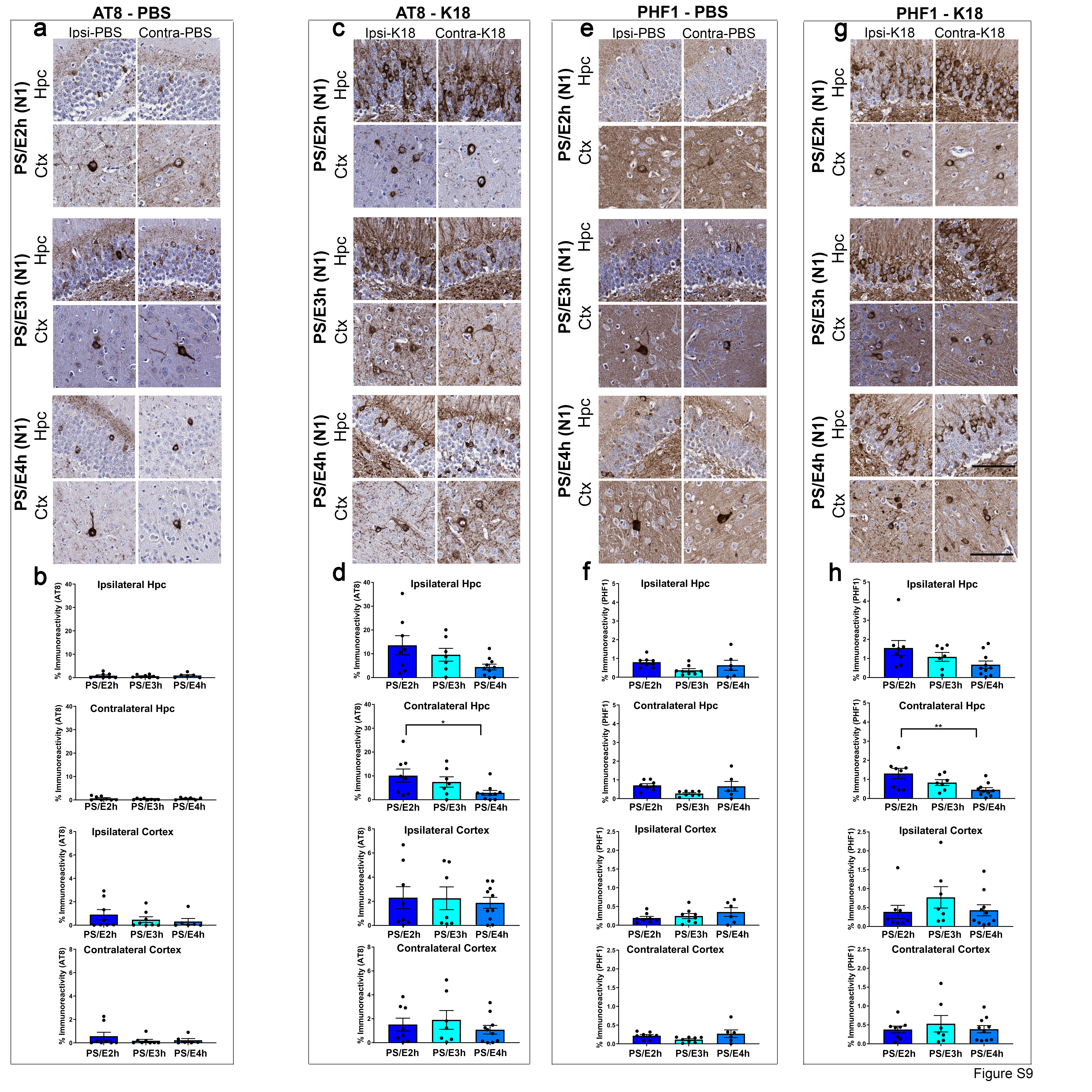

Supplement: Supplementary file 9 — Additional file 9. Figure S9: pau levels in PS19 mice heterozygous for APOE (B6N1 generation) injected with K18-tau aggregates in the hippocampus. K18-tau aggregates or PBS was injected into the left hippocampus of 2.5-month-old PS/E2h, PS/E3h and PS/E4h mice (B6N1 generation) and aged for 5 months. Representative images from the hippocampus (Hpc) and cortex (Ctx) of injected (ipsilateral, ‘IPSI’) and uninjected (contralateral, ‘CONTRA’) hemispheres showing pathology in PS/E2h, PS/E3h and PS/E4h mice. ptau is assessed by AT8 (a, b, c, d) and PHF1 antibodies (e, f, g, h). Quantification of % immunoreactivity is presented from cortex (Ctx) or hippocampus (Hpc) of ipsilateral and contralateral hemispheres of K18-tau aggregate (c, d, g, h) or PBS injected (a, b, e, f) mice. n= 7-10 mice/genotype (K18-tau aggregate group); n= 6-8 mice/genotype (PBS group). 1-way ANOVA *p<0.05, **p<0.01. Scale bar: 70 µm. [file 40478_2022_1359_MOESM9_ESM.jpg]

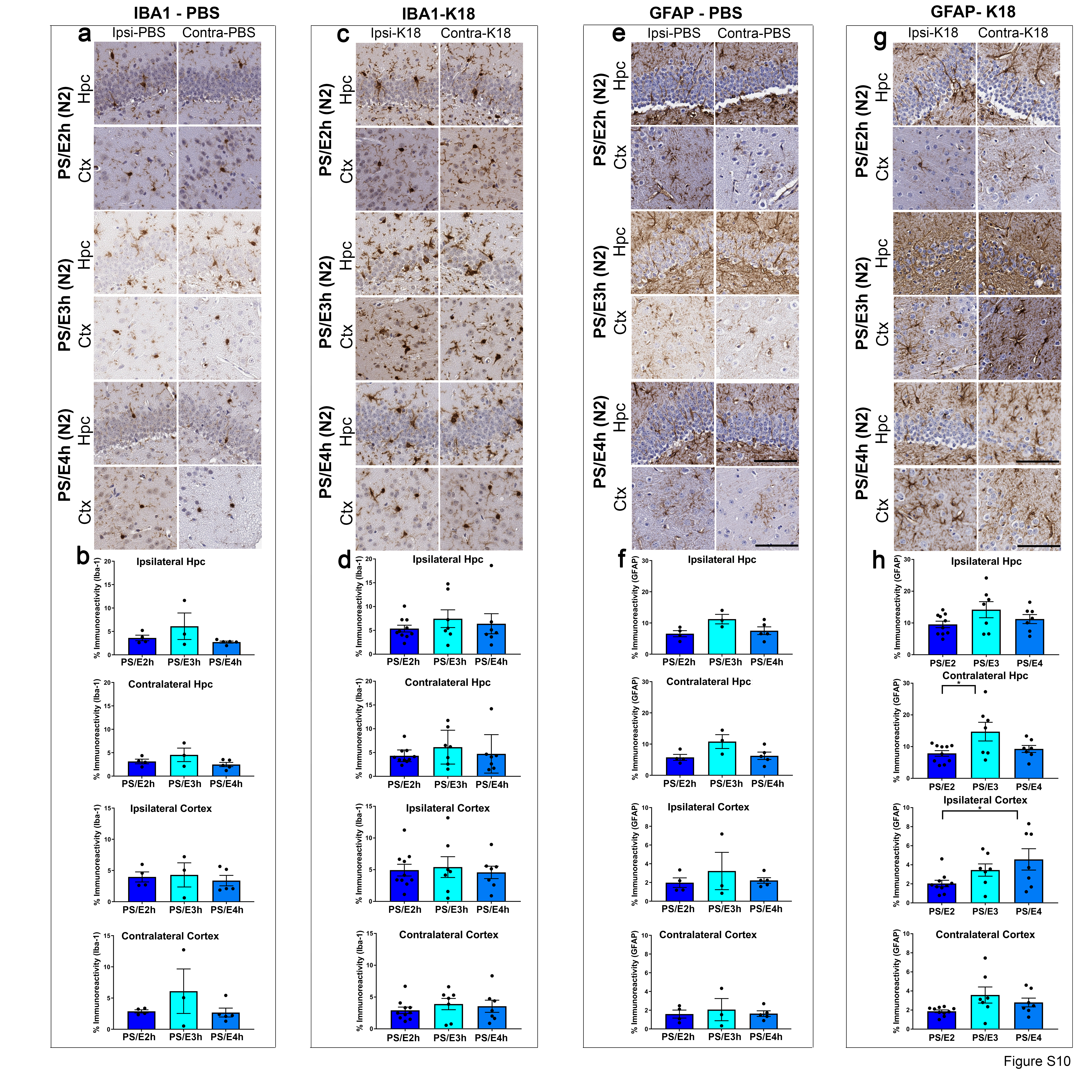

Supplement: Supplementary file 10 — Additional file 10. Figure S10: Gliosis in PS19 mice heterozygous for APOE (B6N2 generation) injected with K18-tau aggregates in the hippocampus. K18-tau aggregates or PBS was injected into the left hippocampus of 2.5-month-old PS/E2h, PS/E3h and PS/E4h mice (B6N2 generation) and aged for 5 months. Representative images from the hippocampus (Hpc) and cortex (Ctx) of injected (ipsilateral, ‘IPSI’) and uninjected (contralateral, ‘CONTRA’) hemispheres showing pathology in PS/E2h, PS/E3h and PS/E4h mice. Microgliosis was assessed using Iba-1 antibody (a-d) and astrogliosis was assessed using GFAP antibody (e-h). Quantification of % immunoreactivity is presented from cortex (Ctx) or hippocampus (Hpc) of ipsilateral and contralateral hemispheres of K18-tau aggregate (c, d, g, h) or PBS injected (a, b, e, f) mice. n= 7-10 mice/genotype (K18-tau aggregate group); n= 3-5 mice/genotype (PBS group). 1-way ANOVA *p<0.05, **p<0.01. Scale bar: 70 µm. [file 40478_2022_1359_MOESM10_ESM.jpg]

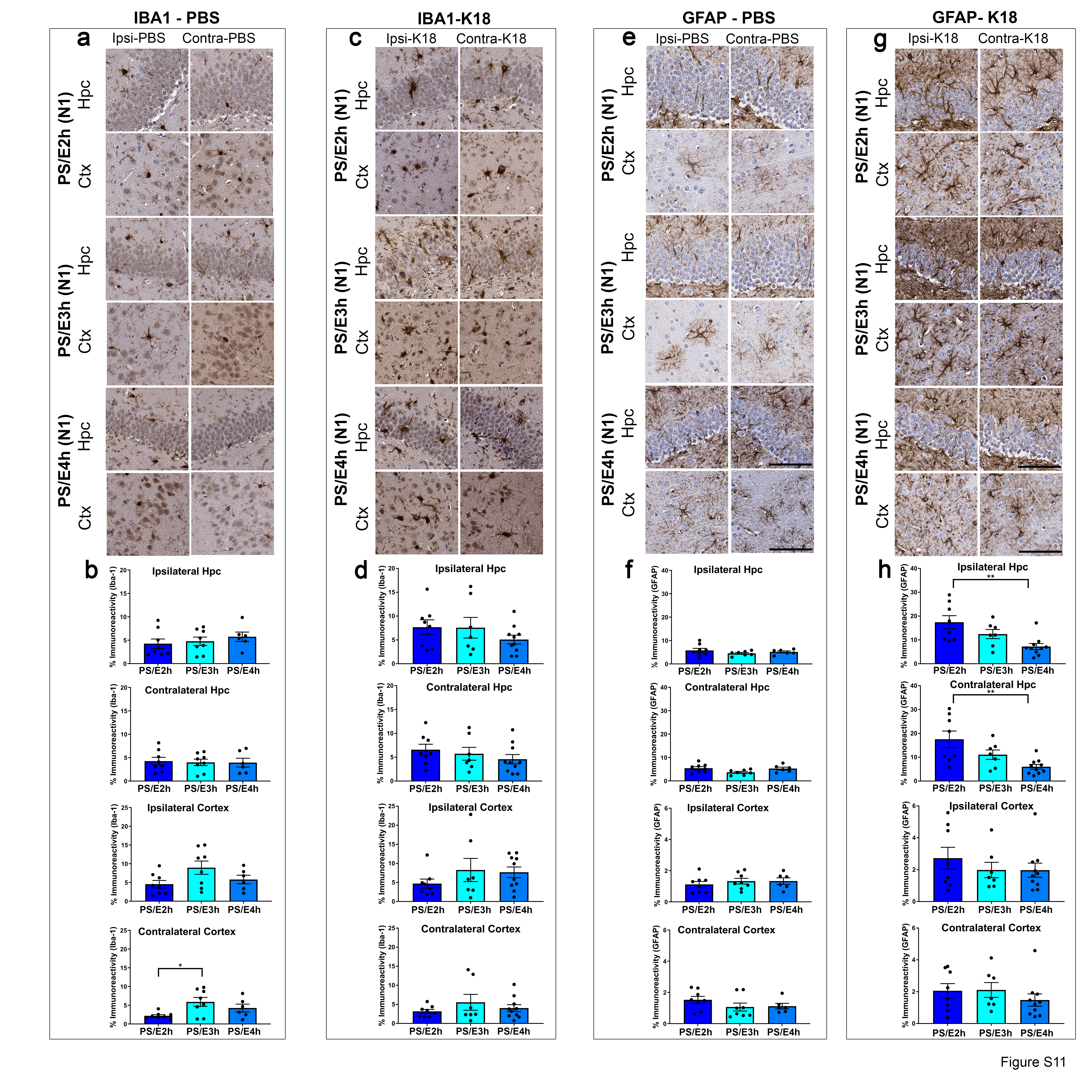

Supplement: Supplementary file 11 — Additional file 11. Figure S11: Gliosis in PS19 mice heterozygous for APOE (B6N1 generation) injected with K18-tau aggregates in the hippocampus. K18-tau aggregates or PBS was injected into the left hippocampus of 2.5-month-old PS/E2h, PS/E3h and PS/E4h mice (B6N1 generation) and aged for 5 months. Representative images from the hippocampus (Hpc) and cortex (Ctx) of injected (ipsilateral, ‘IPSI’) and uninjected (contralateral, ‘CONTRA’) hemispheres showing pathology in PS/E2h, PS/E3h and PS/E4h mice. Microgliosis was assessed using Iba-1 antibody (a-d) and astrogliosis was assessed using GFAP antibody (e-h). Quantification of % immunoreactivity is presented from cortex (Ctx) or hippocampus (Hpc) of ipsilateral and contralateral hemispheres of K18-tau aggregate (c, d, g, h) or PBS injected (a, b, e, f) mice. n= 7-10 mice/genotype (K18-tau aggregate group); n= 6-8 mice/genotype (PBS group). 1-way ANOVA *p<0.05, **p<0.01. Scale bar: 70 µm. [file 40478_2022_1359_MOESM11_ESM.jpg]
