# Supplementary material for: Impact of APOE genotype on prion-type propagation of tauopathy
Source: Acta Neuropathol Commun. 2022 Apr 19;10:57. doi: 10.1186/s40478-022-01359-y (PMC9019935; doi:10.1186/s40478-022-01359-y)
Supplement: Supplementary file 12 — Additional file 12. Table S1:. Description of sample numbers and sex distribution within study cohorts. Table S2. List of antibodies used in the study. Table S3. Table summarizing overall antibody and histological staining data. Table S4. P-values for IHC data obtained APOE homozygous (B6N2) mice injected with K18-tau or PBS. Table S5. P-values for IHC data obtained from APOE heterozygous (B6N2) mice injected with K18-tau or PBS. Table S6. P-values for IHC data obtained from APOE heterozygous (B6N1) mice injected with K18-tau or PBS. Table S7. P-values of AT8 IHC (spinal cord) from APOE homozygous (B6N2) mice injected with K18-tau or PBS. [file 40478_2022_1359_MOESM12_ESM.pdf]

Table S1. Description of sample numbers and sex distribution within study cohorts.

	**PS/E2H (B6N2)**				**PS/E3H (B6N2)**				**PS/E4H (B6N2)**				**PS19**			
	K18		PBS		K18		PBS		K18		PBS		K18		PBS	
Analysis (Antibody)	Male	Female	Male	Female	Male	Female	Male	Female	Male	Female	Male	Female	Male	Female	Male	Female
AT8	5	4	2	3	8	4	2	1	4	5	2	3	6	3	4	5
PHF1	5	4	2	3	8	4	2	1	4	5	2	3	6	3	4	5
TauC3	5	3	2	3	7	3	2	1	3	5	1	2	nd	nd	nd	nd
MC1	5	4	2	3	8	4	2	1	4	5	2	3	6	3	4	5
Iba-1	5	4	2	3	8	4	2	1	4	5	2	3	6	3	4	5
GFAP	5	4	2	3	8	4	2	1	4	5	2	3	6	3	4	5
CD68	5	3	2	2	6	3	2	1	4	5	1	2	nd	nd	nd	nd
Tmem119	5	4	2	3	6	3	2	1	2	5	2	3	nd	nd	nd	nd
Gallyas Silver	3	4	2	2	6	2	2	1	2	4	2	3	5	3	4	5

	**PS/E2h (B6N2)**				**PS/E3h (B6N2)**				**PS/E4h (B6N2)**			
	K18		PBS		K18		PBS		K18		PBS	
Analysis (Antibody)	Male	Female	Male	Female	Male	Female	Male	Female	Male	Female	Male	Female
AT8	4	6	1	3	4	3	1	2	4	3	3	2
PHF1	4	6	1	3	4	3	1	2	4	3	3	2
MC1	4	6	1	3	4	3	1	2	4	3	3	2
Iba-1	4	6	1	3	4	3	1	2	4	3	3	2
GFAP	4	6	1	3	4	3	1	2	4	3	3	2

	**PS/E2h (B6N1)**				**PS/E3h (B6N1)**				**PS/E4h (B6N1)**			
	K18		PBS		K18		PBS		K18		PBS	
Analysis (Antibody)	Male	Female	Male	Female	Male	Female	Male	Female	Male	Female	Male	Female
AT8	5	3	3	5	5	2	3	5	4	6	4	2
PHF1	5	3	3	5	5	2	3	5	4	6	4	2
MC1	5	3	3	5	5	2	3	5	4	6	4	2
Iba-1	5	3	3	5	5	2	3	5	4	6	4	2
GFAP	5	3	3	5	5	2	3	5	4	6	4	2
